# Diversification of Type VI Secretion System Toxins Reveals Ancient Antagonism among Bee Gut Microbes

**DOI:** 10.1128/mBio.01630-17

**Published:** 2017-12-12

**Authors:** Margaret I. Steele, Waldan K. Kwong, Marvin Whiteley, Nancy A. Moran

**Affiliations:** a Microbiology Graduate Program, University of Texas at Austin, Austin, Texas, USA; b Department of Integrative Biology, University of Texas at Austin, Austin, Texas, USA; c Department of Botany, University of British Columbia, Vancouver, BC, Canada; d Department of Molecular Biosciences, University of Texas at Austin, Austin, Texas, USA; e John Ring LaMontagne Center for Infectious Disease, University of Texas at Austin, Austin, Texas, USA; University of California, Berkeley

**Keywords:** *Apis mellifera*, *Bombus*, Rhs toxin, *Snodgrassella alvi*, coevolution

## Abstract

Microbial communities are shaped by interactions among their constituent members. Some Gram-negative bacteria employ type VI secretion systems (T6SSs) to inject protein toxins into neighboring cells. These interactions have been theorized to affect the composition of host-associated microbiomes, but the role of T6SSs in the evolution of gut communities is not well understood. We report the discovery of two T6SSs and numerous T6SS-associated Rhs toxins within the gut bacteria of honey bees and bumble bees. We sequenced the genomes of 28 strains of *Snodgrassella alvi*, a characteristic bee gut microbe, and found tremendous variability in their Rhs toxin complements: altogether, these strains appear to encode hundreds of unique toxins. Some toxins are shared with *Gilliamella apicola*, a coresident gut symbiont, implicating horizontal gene transfer as a source of toxin diversity in the bee gut. We use data from a transposon mutagenesis screen to identify toxins with antibacterial function in the bee gut and validate the function and specificity of a subset of these toxin and immunity genes in *Escherichia coli*. Using transcriptome sequencing, we demonstrate that *S. alvi* T6SSs and associated toxins are upregulated in the gut environment. We find that *S. alvi* Rhs loci have a conserved architecture, consistent with the C-terminal displacement model of toxin diversification, with Rhs toxins, toxin fragments, and cognate immunity genes that are expressed and confer strong fitness effects *in vivo*. Our findings of T6SS activity and Rhs toxin diversity suggest that T6SS-mediated competition may be an important driver of coevolution within the bee gut microbiota.

## IMPORTANCE

The structure and composition of host-associated bacterial communities are of broad interest, because these communities affect host health. Bees have a simple, conserved gut microbiota, which provides an opportunity to explore interactions between species that have coevolved within their host over millions of years. This study examined the role of type VI secretion systems (T6SSs)—protein complexes used to deliver toxic proteins into bacterial competitors—within the bee gut microbiota. We identified two T6SSs and diverse T6SS-associated toxins in bacterial strains from bees. Expression of these genes is increased in bacteria in the bee gut, and toxin and immunity genes demonstrate antibacterial and protective functions, respectively, when expressed in *Escherichia coli*. Our results suggest that coevolution among bacterial species in the bee gut has favored toxin diversification and maintenance of T6SS machinery, and demonstrate the importance of antagonistic interactions within host-associated microbial communities.

## INTRODUCTION

Host-associated microbiota are often complex communities comprised of hundreds of species. Bacteria that live in these communities employ diverse mechanisms of intercellular competition. Though first identified as pathogenicity factors (1), type VI secretion systems (T6SSs) are increasingly recognized for their role in mediating antagonistic interactions between bacteria (2). These multiprotein complexes participate in contact-dependent intercellular competition by driving needle-like structures, which can be loaded with a variety of toxins, through the membranes of nearby cells (3, 4). T6SSs have been shown to contribute to spatial organization of bacterial communities *in vitro* (5, 6). Furthermore, recent studies have shown that human gut microbes utilize T6SSs in interbacterial antagonism *in vitro* and in gnotobiotic mouse models; these findings suggest roles for these complexes in the cocolonization and persistence of bacterial species in the human gut (7–10). Although T6SSs are common—present in approximately 25% of sequenced genomes of Gram-negative bacteria (4, 11)—little is known about the role of T6SSs in the evolution of commensal communities.

The Western honey bee, *Apis mellifera*, has a highly conserved gut microbiota with properties comparable to those of mammalian gut communities, including high strain diversity, social transmission, and conferral of benefits to host health (12–15). The core gut community is comprised of nine bacterial species, which account for more than 95% of bacteria in the guts of healthy worker bees (16). We previously found T6SS and T6SS-associated effector genes in the genomes of some of these species, including the betaproteobacterium *Snodgrassella alvi* and gammaproteobacterium *Gilliamella apicola* (17). Therefore, T6SS-mediated competition among coevolved species may influence the structure and composition of the bee gut microbiota, as has been hypothesized for the gut communities of mammals based on bioinformatic analyses and on patterns of antimicrobial antagonism *in vitro* and in gnotobiotic mice (7–9, 11).

*S. alvi* is an abundant gut symbiont of honey bees (*Apis* spp.) and their close relatives, the bumble bees (*Bombus* spp.). It primarily colonizes the ileum section of the hindgut, where it forms biofilm-like layers with *G. apicola* (16, 18). Multiple *S. alvi* strains coexist within individual bees and bee hives, and strains differ among host species and geographic locations (13, 16, 19). How such strain diversity arises and is maintained in gut microbiomes is unclear (20). However, it is likely that interactions between members of the microbiota affect strain-level composition, which in turn may influence community-scale trends in stability, turnover, and diversity (21).

We hypothesize that T6SSs and T6SS-associated toxins mediate intraspecific competition among *S. alvi* strains in the bee gut, as well as interspecific competition between *S. alvi* and other gut microbes. We used transcriptome sequencing (RNA-Seq) to determine the conditions under which *S. alvi* T6SSs are expressed. To examine the diversity, prevalence, and evolution of T6SSs and their associated toxins in this gut symbiont, we isolated and sequenced the genomes of 28 *S. alvi* strains from diverse *Apis* and *Bombus* species. Finally, we provide evidence that T6SS-associated Rhs toxins have antibacterial activity *in vivo* and that extensive recombination and horizontal transfer of toxin/immunity genes between strains in the microbiota have resulted in tremendous diversity in their toxin repertoires. Our results support the view of gut microbiomes as exclusive assemblages whose membership is influenced by complex competitive interactions among coevolving species.

## RESULTS

### *S. alvi* upregulates T6SSs *in vivo.*

**FIG 1:**
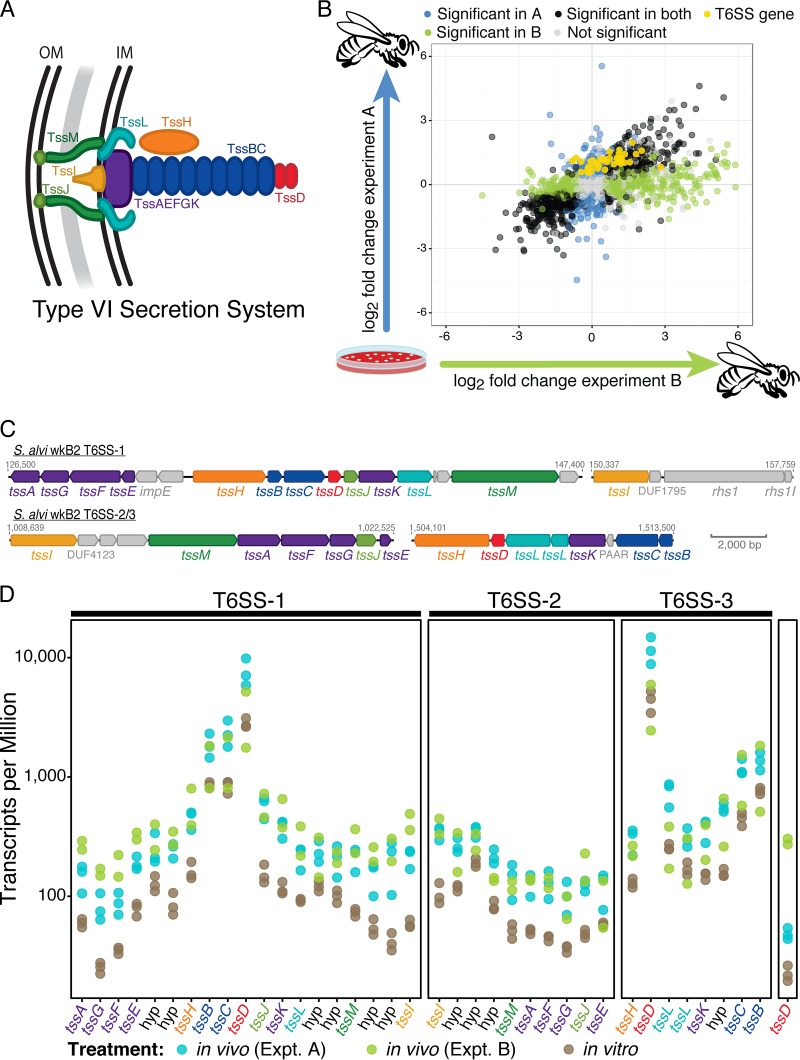
Genes involved in intercellular competition are upregulated *in vivo*. (A) Diagram of the T6SS, showing the membrane complex (TssJ, TssL, and TssM; green), baseplate complex (TssA, TssE, TssF, TssG, and TssK; purple), a TssD needle (red) tipped with a TssI spike (yellow), the contractile sheath (TssB and TssC; blue), and an ATPase (TssH; orange). (B) *In vivo* fold change in each experiment for all genes. Axes depict comparisons between experimental treatments, with the *in vitro* condition serving as the control. Experiment A, from the work of Powell et al. (23); experiment B, from this study. T6SS-associated genes are highlighted in yellow. (C) ORF maps of the three T6SS loci in *S. alvi* wkB2; genes encoding core components are colored to match panel A, and gray numbers indicate the position of the diagrammed region within the genome. (D) Count-normalized gene expression of T6SS-associated genes for *in vivo* and *in vitro* replicates.

Inspection of the genome of the *S. alvi* type strain, wkB2, revealed 38 T6SS-associated genes clustered at three genomic loci. One locus (T6SS-1) contains 19 genes, including all 13 T6SS core components (17), while two other loci (T6SS-2 and T6SS-3 or, collectively, T6SS-2/3) contain complementary sets of 9 and 8 genes that together encode a second T6SS with very little amino acid sequence identity (17.3% average) to the genes in T6SS-1 (Fig. 1A and C). From this, we predict that two complete T6SS complexes are encoded in the *S. alvi* wkB2 genome. In addition to the core T6SS genes, the T6SS-1 locus contains *impE* and five hypothetical genes, while the T6SS-2/3 loci include four hypothetical genes, including a gene encoding a proline-alanine-alanine-arginine (PAAR) domain and a gene encoding a protein with a DUF4123 domain. This DUF4123 domain protein is likely to be an effector chaperone that facilitates interaction between an effector and the T6SS-2/3 VgrG (22).

To determine whether the T6SSs identified in *S. alvi* wkB2 are expressed *in vivo*, we used whole-transcriptome sequencing (RNA-Seq) to measure gene expression of *S. alvi* wkB2 in gnotobiotic *A. mellifera* workers. To account for variation due to host genetic background, we also reanalyzed a published RNA-Seq data set—produced with the same experimental design—from the work of Powell et al. (23) and compared gene expression in each *in vivo* experiment to expression under standard laboratory culture. We identified 583 genes—including 19 T6SS genes—that were differentially expressed in the same direction in these two independent *in vivo* RNA-Seq experiments, relative to *in vitro* culture (Fig. 1B). Of the 19 T6SS genes that were upregulated *in vivo* in both experiments, 13 were from T6SS-1, 5 were from T6SS-2/3, and one (*tssD*) was not part of a T6SS locus (Fig. 1D). An additional 12 T6SS genes were significantly upregulated in only the data set of Powell et al. (see Table S1 in the supplemental material).

10.1128/mBio.01630-17.5 TABLE S1  Differential expression of *S. alvi* wkB2 T6SS genes, Rhs toxin and putative immunity genes, and genes involved in iron and nitrogen metabolism. Download TABLE S1, DOCX file, 0.2 MB. Copyright © 2017 Steele et al. 2017 Steele et al. This content is distributed under the terms of the Creative Commons Attribution 4.0 International license.

### *S. alvi* T6SSs are vertically inherited.

**FIG 2:**
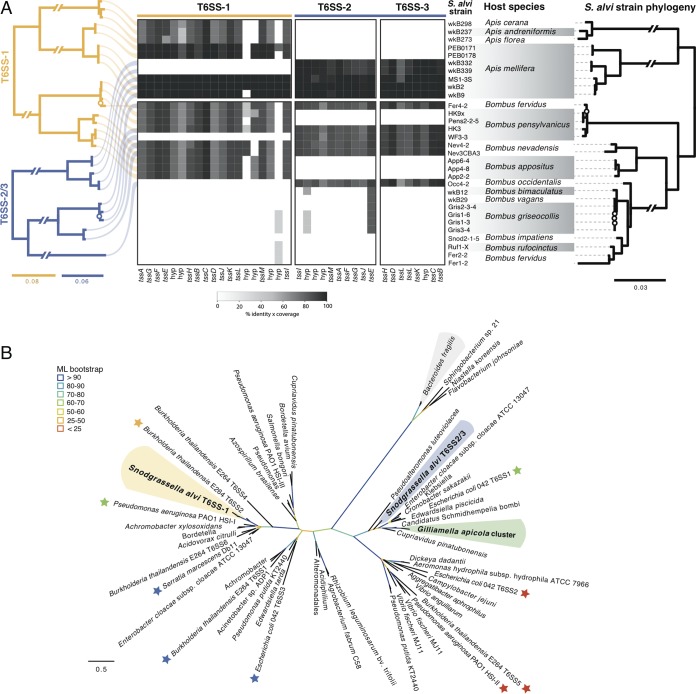
Two T6SSs are present in the bee gut symbiont *S. alvi*. (A) Presence of T6SS genes in *S. alvi* strains based on similarity to genes of the three T6SS loci of strain wkB2; the heat map shows the percent identity × coverage of the best match in each genome to the corresponding wkB2 gene. The *S. alvi* phylogeny (right) is reconstructed from ribosomal protein genes; nodes with less than 80% bootstrap support are marked with an open circle. The yellow and blue trees (left) show relationships between T6SS-1 and T6SS-2/3 loci, respectively. hyp, hypothetical open reading frame. (B) Maximum likelihood tree for TssB showing the relationship between T6SS-1 and T6SS-2/3. Sequences from T6SS with characterized functions are marked with stars: pathogenesis (red), intercellular competition (blue), Rhs-associated (yellow), and secretion of antimicrobials (green).

To better understand the evolution and ecology of T6SSs within *S. alvi*, we sequenced the genomes of 28 *S. alvi* strains isolated from diverse *Apis* and *Bombus* species collected from Southeast Asia and North America (Table S2). We determined whether T6SSs were present in these strains, as well as in three previously published strains (17), by screening for homologs of the T6SS genes in *S. alvi* wkB2. All seven *S. alvi* strains isolated from *A. mellifera* encoded at least one complete T6SS complex, and only the two strains from bees collected in Malaysia (wkB332 and wkB339) were missing T6SS-1 (Fig. 2A). *S. alvi* strains isolated from three honey bee species (*Apis andreniformis*, *Apis cerana*, and *Apis florea*) collected in Singapore also encoded T6SS-1. Both T6SS-1 and T6SS-2/3 were present in strains isolated from *Bombus pensylvanicus*, *Bombus nevadensis*, and *Bombus appositus*, although some strains lacked one or the other system. Finally, 10 strains isolated from seven different *Bombus* host species appear to have lost both T6SSs. To ensure that our homology-based searches had not missed any T6SS loci with low identity to T6SS-1 and T6SS-2/3, we searched for the 13 core T6SS genes in the genome annotations generated by the Rapid Annotation using Subsystems Technology (RAST) pipeline (24) for all 31 strains. We did not find any additional T6SS loci, confirming that only these two subfamilies of T6SSs are present in the entire set of *S. alvi* strains.

10.1128/mBio.01630-17.6 TABLE S2  Bacterial strains and genomes used in this study. Download TABLE S2, DOCX file, 0.1 MB. Copyright © 2017 Steele et al. 2017 Steele et al. This content is distributed under the terms of the Creative Commons Attribution 4.0 International license.

We reconstructed the phylogenetic relationships of the *S. alvi* strains using 37 single-copy ribosomal protein genes (Table S3), as well the relationships of the two T6SSs based on their constituent genes (Fig. 2A). The phylogenies of T6SS-1 and T6SS-2/3 were both highly congruent with the ribosomal protein-gene phylogeny, suggesting that evolution of *S. alvi* T6SSs occurs mainly by vertical descent and not through transfers between strains. Further, this phylogenetic congruence supports the acquisition of both T6SSs prior to the divergence of the *S. alvi* clades associated with *Apis* and *Bombus* hosts, with subsequent losses leading to the pattern of T6SS presence/absence observed across our strains (Fig. 2A and S1).

10.1128/mBio.01630-17.1 FIG S1  Ongoing deletion is eliminating the T6SS-2 locus in some *S. alvi* strains. The T6SS-2 locus in *S. alvi* strains PEB0171, wkB2, HK9, Gris1-3, and Fer1-2, which is bracketed by *recO* and integration host factor α in all but wkB2. The *S. alvi* strain phylogeny (left) shows the relationships between strains and supports the hypothesis that T6SS-2 has been lost multiple times in *S. alvi*. Download FIG S1, PDF file, 0.1 MB. Copyright © 2017 Steele et al. 2017 Steele et al. This content is distributed under the terms of the Creative Commons Attribution 4.0 International license.

10.1128/mBio.01630-17.7 TABLE S3  *S. alvi* genes used to reconstruct strain phylogeny and genes used for tests of positive selection. Download TABLE S3, DOCX file, 0.1 MB. Copyright © 2017 Steele et al. 2017 Steele et al. This content is distributed under the terms of the Creative Commons Attribution 4.0 International license.

The phylogeny of the T6SS TssB protein was reconstructed to determine the relatedness of the *S. alvi* T6SSs to previously identified T6SSs. We found that TssB proteins associated with *S. alvi* T6SS-1 and T6SS-2/3 are phylogenetically distinct and have different evolutionary origins (Fig. 2B). TssB from *S. alvi* T6SS-1 clusters with proteins from *Pseudomonas aeruginosa* PAO1 HSI-I and *Burkholderia thailandensis* T6SS-2 and T6SS-6, while TssB from *S. alvi* T6SS-2/3 is more closely related to proteins from other Gram-negative bee symbionts, including *G. apicola*, *Frischella perrara*, and “*Candidatus* Schmidhempelia bombi,” and other gammaproteobacteria.

### T6SS-1 likely mediates secretion of diverse Rhs toxins.

T6SSs secrete a variety of antibacterial proteins, including the Rhs family of toxins (25). We previously found numerous Rhs toxins encoded in the genomes of *A. mellifera* gut symbionts, including *S. alvi* wkB2 (17). Many of the newly sequenced genomes also contain large numbers of Rhs genes—up to 120 copies in some strains—but the function of these genes in the ecology of the bee gut is unclear.

**FIG 3:**
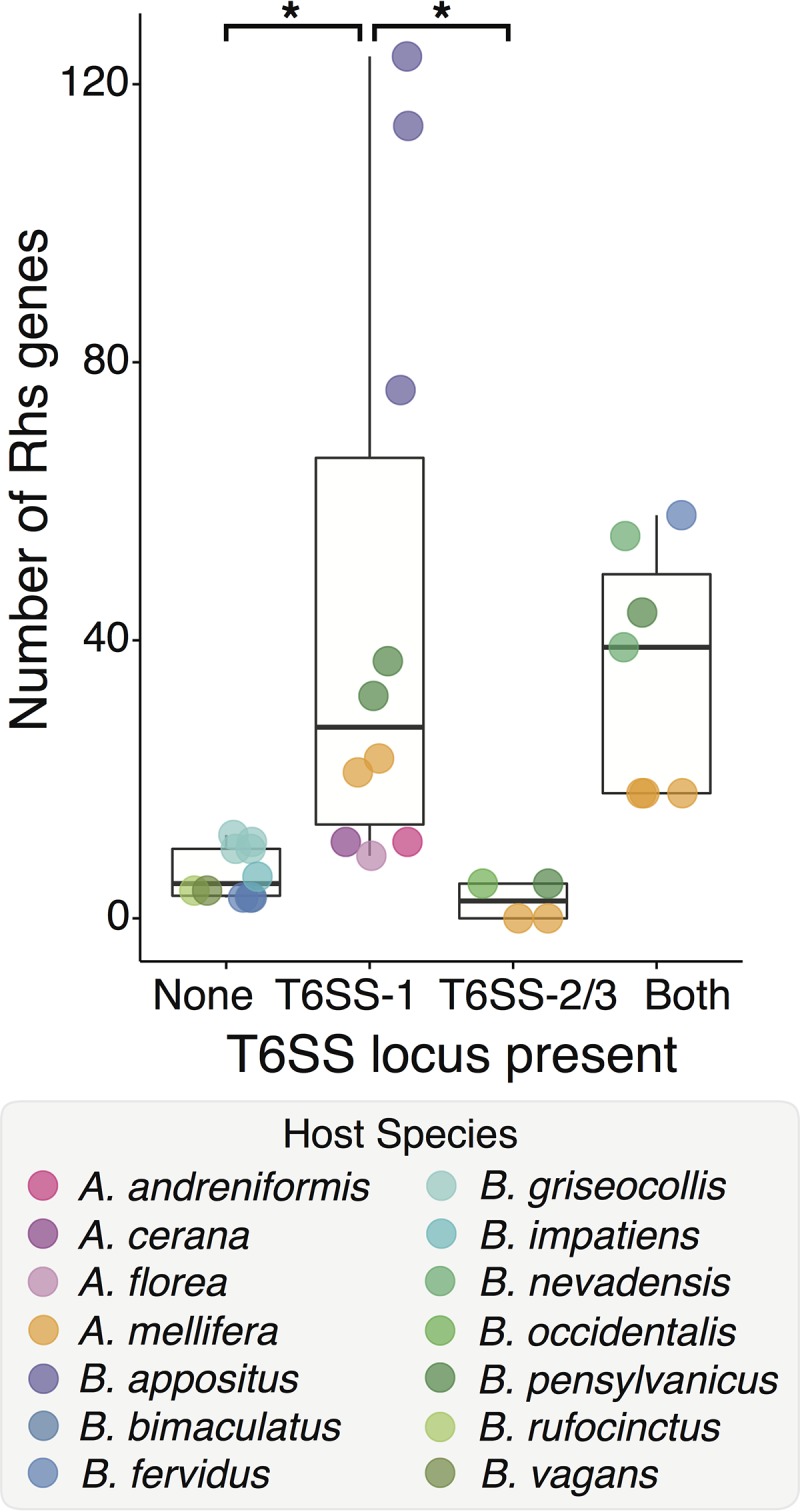
Rhs genes are associated with T6SS-1. The number of Rhs-family genes is significantly higher in genomes with the T6SS-1 locus, regardless of the presence or absence of the T6SS-2/3 locus (one-way ANOVA with Tukey’s HSD multiple-test correction; *, *P*_adj_ < 0.05).

We tested whether *S. alvi* Rhs toxins were associated with a particular T6SS locus and found that strains encoding T6SS-1 harbored significantly more Rhs genes than strains with no T6SS or only T6SS-2/3 (Fig. 3). In contrast, the number of Rhs genes per genome did not vary significantly with the presence or absence of T6SS-2/3. Among strains encoding T6SS-1, strains isolated from *Bombus* had more Rhs genes than did strains from *Apis*. However, the correlation between the presence of T6SS-1 and the number of Rhs toxins persisted when strains from *Apis* or *Bombus* hosts were examined separately (Fig. S2). Furthermore, the T6SS-1 *tssI* (*vgrG*) gene is located at the 3′ end of the T6SS locus, immediately upstream of one of the three Rhs loci in wkB2, while the T6SS-2/3 *tssI* gene occurs at the 5′ end of the locus, upstream of genes encoding proteins of unknown function (Fig. 1C). This strongly implicates a role for T6SS-1 in Rhs toxin secretion while suggesting that other, unidentified effectors are secreted through T6SS-2/3.

10.1128/mBio.01630-17.2 FIG S2  The association between *S. alvi* Rhs genes and T6SS-1 is consistent across host species. Statistical tests performed with ANOVA and Tukey’s HSD. *, *P*_adj_ < 0.05; ***, *P*_adj_ < 0.005. Download FIG S2, PDF file, 0.4 MB. Copyright © 2017 Steele et al. 2017 Steele et al. This content is distributed under the terms of the Creative Commons Attribution 4.0 International license.

### Rhs loci have a characteristic architecture.

**FIG 4:**
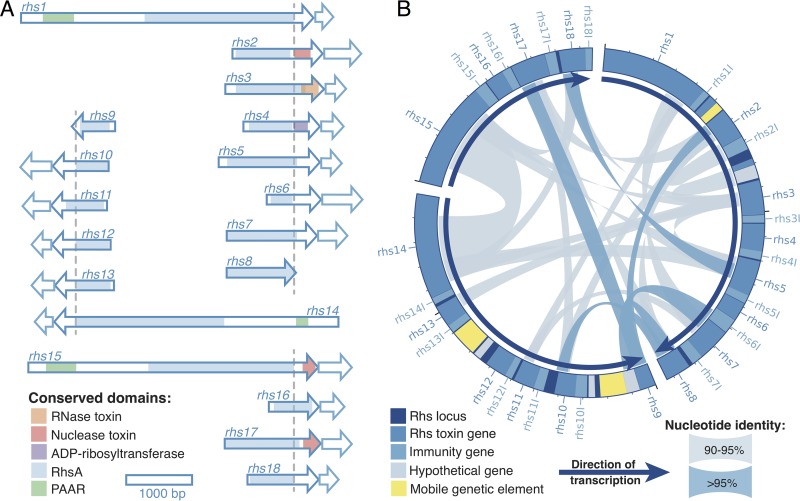
*S. alvi* wkB2 has three Rhs loci with architectures congruent with extensive toxin domain replacement. (A) Size and structure of *S. alvi* wkB2 Rhs genes and cognate immunity genes, shown as arrows outlined in dark and light blue, respectively. A dashed line indicates the location of the conserved DPXG(18)DPXG motif, which marks the start of the C-terminal toxin domain in each toxin. PAAR, RhsA, and toxin domains were predicted through the NCBI CDD search (E value, <10^−5^). (B) Arrangement of Rhs loci in the wkB2 genome. Arrows show the 5′-to-3′ orientation of the loci, while dark and light blue ribbons connect >500-bp regions with >95% and >90% nucleotide identity, respectively. Rhs and cognate immunity genes are highlighted in dark and light blue, respectively; putative mobile genetic elements are yellow; and hypothetical genes are gray. Major tick marks occur every 10,000 bp; minor tick marks occur every 1,000 bp.

We examined the organization of Rhs loci in the completely closed genome of *S. alvi* wkB2 to better understand the structural diversity of these putative T6SS effectors. We identified 18 Rhs-family genes at three separate loci in wkB2. Rhs-family genes encode large, polymorphic proteins with variable C-terminal toxin domains and conserved core regions that contain the Rhs/YD repeats characteristic of this toxin family (26). Though many T6SSs secrete cargo effectors that form noncovalent associations with secreted components of the T6SS, Rhs toxins are often specialized effectors, fused to PAAR domains that interact with VgrG at the tip of the T6SS needle (25, 27). In contrast to cargo effectors, which are often classified by their cellular targets, the Rhs toxin family is defined by a core region containing characteristic Rhs repeats and includes proteins with variable C-terminal toxin domains that affect a variety of cellular targets within prokaryotes and eukaryotes (26). The highly conserved DPXG(18)DPXG motif, which is found at the end of the conserved core region (26), was used to predict the start of the C-terminal toxin domain in each Rhs gene, and the Conserved Domain Database (CDD) (28) was used to search for similarity to previously characterized toxin domains. Three genes (*rhs1*, *rhs14*, and *rhs15*) encode proteins with large N-terminal regions that contain complete RhsA domains (COG3209) and PAAR secretion domains (Fig. 4A). Each of these genes is found at the 5′ end of its respective Rhs locus and is followed by several relatively short Rhs genes with intact core and toxin domains, truncated RhsA domains, and no PAAR domain (Fig. 4B). Based on conserved motifs, Rhs8 and Rhs9, which are encoded at the 3′ end of their respective Rhs loci, appear to have truncated N-terminal domains and no C-terminal toxin domains. The C-terminal regions of five Rhs genes were similar to previously characterized toxin domains, including three domains associated with toxins of the HNH/endonuclease VII family, an RNase toxin domain, and an ADP-ribosyltransferase domain (Table S4), whereas the remaining 11 Rhs proteins may contain novel toxins.

10.1128/mBio.01630-17.8 TABLE S4  Conserved protein domains detected in *S. alvi* wkB2 Rhs toxin genes (E value, <0.00001) and immunity genes (E value, <0.001). Download TABLE S4, DOCX file, 0.1 MB. Copyright © 2017 Steele et al. 2017 Steele et al. This content is distributed under the terms of the Creative Commons Attribution 4.0 International license.

### Rhs toxin genes have antibacterial function and are upregulated *in vivo.*

Like toxin-antitoxin systems, Rhs loci typically encode immunity proteins that protect the cell against the activity of their cognate toxins. These immunity genes generally have little sequence homology to each other, making it difficult to infer their activity from sequence data alone, but they are often found immediately downstream of their corresponding toxin (26, 29). We identified putative immunity genes immediately downstream of 16 Rhs toxin genes in the *S. alvi* wkB2 genome (Fig. 4A). Only *rhs8* and *rhs9*, which do not have C-terminal toxin domains, lacked adjacent putative immunity genes.

**FIG 5:**
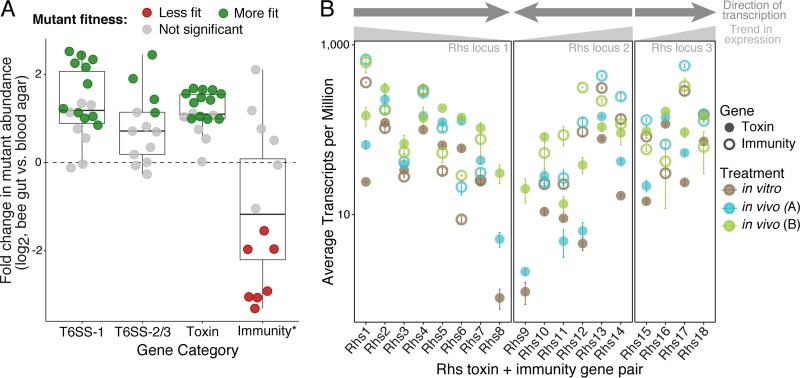
Rhs toxins and immunity genes are upregulated and have antibacterial function *in vivo*. (A) Fold change in the abundance of *S. alvi* wkB2 mutants with transposon insertions in T6SS-associated genes, Rhs toxins, or putative Rhs immunity genes *in vivo*, relative to abundance *in vitro*. DESeq2 (55) was used to determine differential mutant fitness with a false discovery rate cutoff of 0.05. Green and red circles indicate mutants that are significantly more or less fit *in vivo* (*P*_adj_ < 0.05), respectively. Gray circles indicate genes with a *P*_adj_ of >0.05. *, three putative immunity genes that were essential *in vivo* are excluded. Essential genes were identified by comparing the frequency of observed transposon insertions for each gene to the frequency of insertion in a randomized data set, as described by Turner et al. (74). (B) Expression of Rhs toxin and immunity gene pairs, averaged across replicates, in *in vitro* culture and in two *in vivo* experiments: *in vivo* experiment A, from the work of Powell et al. (23); *in vivo* experiment B, from this paper. Gray arrows show the 5′-to-3′ organization of genes for each locus.

To ascertain the functionality of the Rhs toxin-antitoxins in *S. alvi*, we reanalyzed data from a recent genome-wide transposon mutagenesis screen (Tn-Seq) of strain wkB2 (23) using our updated Rhs annotations. We found that three putative immunity genes were completely intolerant to disruption by transposon insertion, suggesting that these genes are essential (Fig. 5B; Table S5). Insertions in a further seven putative immunity genes were significantly detrimental to the fitness of *S. alvi* wkB2 in the bee gut, which demonstrates the importance of Rhs immunity genes for fitness within the host and indicates that the corresponding Rhs toxins in *S. alvi* do indeed have antibacterial activity. Some of the Rhs toxin genes whose cognate immunity genes do not contribute to fitness may not encode functional proteins. For example, *rhs3* has been pseudogenized by multiple nonsense mutations, while *rhs6* and *rhs16* have truncated N-terminal regions that do not prevent their transcription but may interfere with their activity. Alternatively, these toxins may target species other than *S. alvi*.

10.1128/mBio.01630-17.9 TABLE S5  Fitness effects of transposon insertions in *S. alvi* wkB2 T6SS, Rhs toxin, and putative immunity genes. Download TABLE S5, DOCX file, 0.1 MB. Copyright © 2017 Steele et al. 2017 Steele et al. This content is distributed under the terms of the Creative Commons Attribution 4.0 International license.

**FIG 6:**
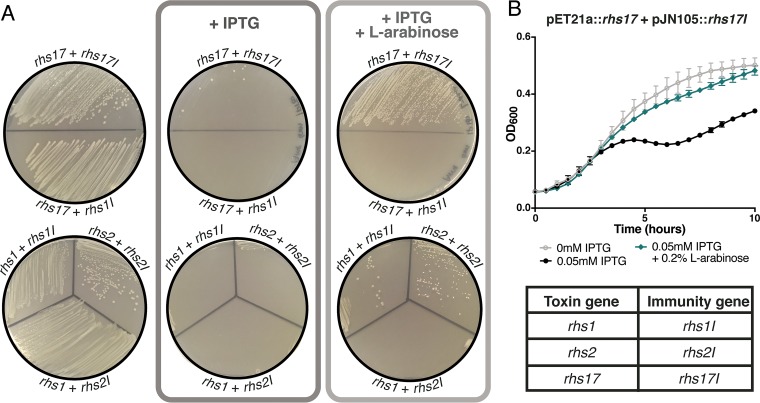
Expression of cognate immunity genes restores growth in cells expressing Rhs toxins. (A) *E. coli* BL21(DE3) cells containing *rhs17*, *rhs1*, and *rhs2* under the control of the IPTG-inducible promoter on pET21a grow on selective medium in the absence of induction (left) and do not grow when toxin expression is induced with 0.1 mM IPTG (center). Induction of the cognate immunity gene with 0.5% l-arabinose restores growth in the presence of IPTG (right). Induction of *rhs1I* does not restore growth in cells expressing *rhs17*, and *rhs2I* does not restore growth in cells expressing *rhs1*. (B) Induction of *rhs17I* with 0.2% l-arabinose (teal) restores growth in cells with induced toxin expression (black) in liquid culture.

To further verify the function of these genes, we cloned three pairs of toxin and immunity genes into compatible expression vectors in *Escherichia coli* BL21(DE3). The toxin domains of *rhs1*, *rhs2*, and *rhs17* were cloned into the pET21a expression vector under the control of an IPTG (isopropyl-β-d-thiogalactopyranoside)-inducible P_T7_ promoter. The putative immunity genes *rhs1I*, *rhs2I*, and *rhs17I* were cloned into expression vector pJN105 under the control of the l-arabinose-inducible P_BAD_ promoter. Both *rhs1I* and *rhs2I* were identified as essential in the *S. alvi* wkB2 Tn-Seq analysis; *rhs17I* was important for fitness *in vivo*. *E. coli* cells containing pET21a::*rhs17* and pJN105::*rhs17I* were able to grow on lysogeny broth (LB) agar without induction or LB containing 0.1 mM IPTG and 0.5% l-arabinose to induce expression of both toxin and immunity genes, but not on LB containing 0.1 mM IPTG alone, which induces expression of only the toxins (Fig. 6A). Induction of immunity gene expression with l-arabinose did not restore the growth of cells with pJN105::*rhs1I* instead of pJN105::*rhs17I* (Fig. 6A and S3B). Additionally, *E. coli* cells containing *rhs17* and *rhs17I* demonstrate a growth defect in liquid cultures containing 0.05 mM IPTG that is counteracted by the addition of 0.2% l-arabinose (Fig. 6B). The observed growth defect is dependent on the presence of the toxin gene, while the restoration of growth with the addition of l-arabinose occurs only in cells with the cognate immunity gene (Fig. S3A). Similar growth patterns were observed for cells expressing *rhs1* and *rhs1I* or *rhs2* and *rhs2I* (Fig. 6A and S3C and D), though cells containing *rhs1* exhibit much slower growth and often fail to grow, even in the absence of toxin gene induction.

10.1128/mBio.01630-17.3 FIG S3  Expression of the cognate immunity gene restores growth in cells expressing the *rhs1* and *rhs2* toxin genes. (A) l-Arabinose does not counteract the effects of toxin gene induction in the absence of the immunity gene. (B) Similarly, the presence of IPTG does not lead to reduced growth in the absence of the toxin gene. (C) Cells grown with 0.1 mM IPTG to induce *rhs17* expression (dark teal circles and dark purple triangles) exhibit reduced growth relative to cells grown without IPTG (open circles and open triangles). Growth is restored by inducing immunity gene expression with 0.1% l-arabinose in cells containing *rh17I* (light teal circles) but not in cells with *rhs1I* (light purple triangles). (D and E) *E. coli* BL21(DE3) cells expressing *rhs1* (D) or *rhs2* (E) induced by IPTG demonstrate a severe growth defect in liquid culture that is at least partially alleviated by expression of the cognate immunity gene induced by l-arabinose. Download FIG S3, PDF file, 1 MB. Copyright © 2017 Steele et al. 2017 Steele et al. This content is distributed under the terms of the Creative Commons Attribution 4.0 International license.

As our RNA-Seq results indicated that T6SS expression was upregulated *in vivo* (Fig. 1D), we examined whether this also held true for Rhs toxin expression. Fifteen Rhs toxins were significantly upregulated *in vivo* in at least one experiment, and seven of these were significantly upregulated in both experiments (Fig. 5A; Table S1). We also observed a trend in expression, whereby—after normalizing for length—genes located at the 5′ end of the Rhs locus were more transcriptionally active than genes at the 3′ end (Fig. 5A). This trend was highly significant for the toxin genes of Rhs loci 1 and 2 (Spearman’s rank correlation ρ = −0.388, *P* < 0.005, and Spearman’s rank correlation ρ = −0.563, *P* < 0.001, respectively), although this trend was not observed for Rhs locus 3 (Spearman’s rank correlation ρ = 0.375, *P* = 0.034).

### A large pool of interchangeable toxins drives Rhs ecology.

**FIG 7:**
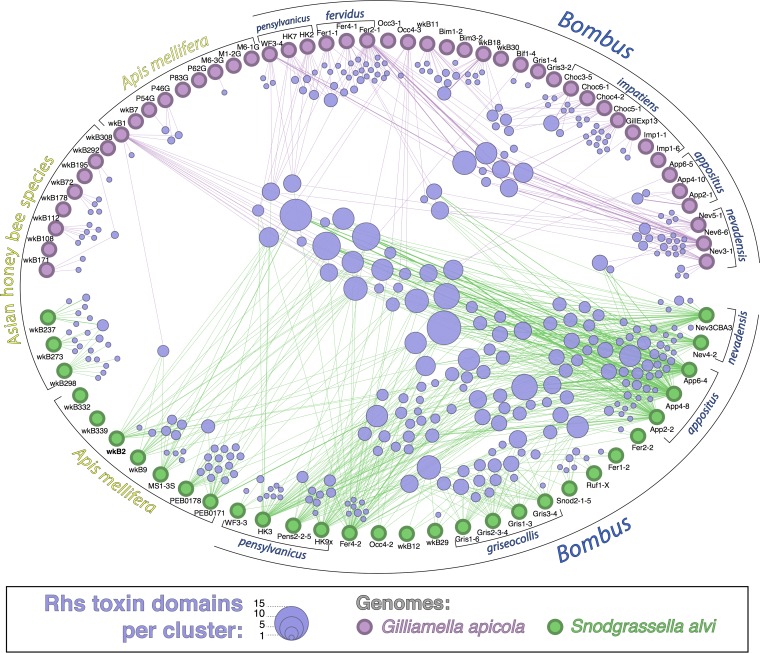
Members of the bee gut microbiome encode diverse Rhs toxins, which are shared within and between species. Each toxin node represents a unique C-terminal toxin domain derived by clustering at 90% nucleotide identity; size is proportional to number of sequences in a cluster. Lines connect toxin nodes to strains in which they are found. Strains are grouped and labeled according to host of origin. Spatial configuration of the Rhs clusters is to minimize overlap of lines and circles for visibility and does not correspond to any other metadata. Strain names are provided adjacent to each strain symbol.

The large number of Rhs toxins encoded by *S. alvi* strains suggests that toxin diversity may be important for competitive ability among members of the bee gut microbiota. We examined the diversity and prevalence of these genes in all *S. alvi* strains, as well as in strains of a common coresident gut bacterium, *G. apicola*. In the 77 genomes analyzed (31 of *S. alvi* and 46 of *G. apicola*), we detected a total of 1,112 Rhs genes (813 from *S. alvi* and 299 from *G. apicola*), encoding potentially 364 distinct toxin domains. This reveals a tremendous pool of Rhs diversity accessible by, and possibly unique to, the bee gut microbiome (Fig. 7). Some toxins are found in both *S. alvi* and *G. apicola*, while others appear to be constrained to a single species or a set of closely related strains.

In contrast to the T6SS structural genes, Rhs toxin distribution is not solely determined by vertical descent. Clustering toxin domains by cooccurrence reveals networks of dissemination that are likely governed by geography and shared host species. For instance, strains from Southeast Asia did not share any toxins with those from North America. Host relatedness does not pose a complete barrier, as *S. alvi* strains from both *Apis* and *Bombus* hosts carried many toxins in common. Toxins are mostly shared within a species (*S. alvi* or *G. apicola*) rather than between species, which may reflect more frequent gene transfer between conspecific bacterial strains or indicate that this toxin family is predominantly used for intraspecific, rather than interspecific, competition in these two gut symbionts.

### Rhs toxin and immunity gene pairs are strongly linked.

**FIG 8:**
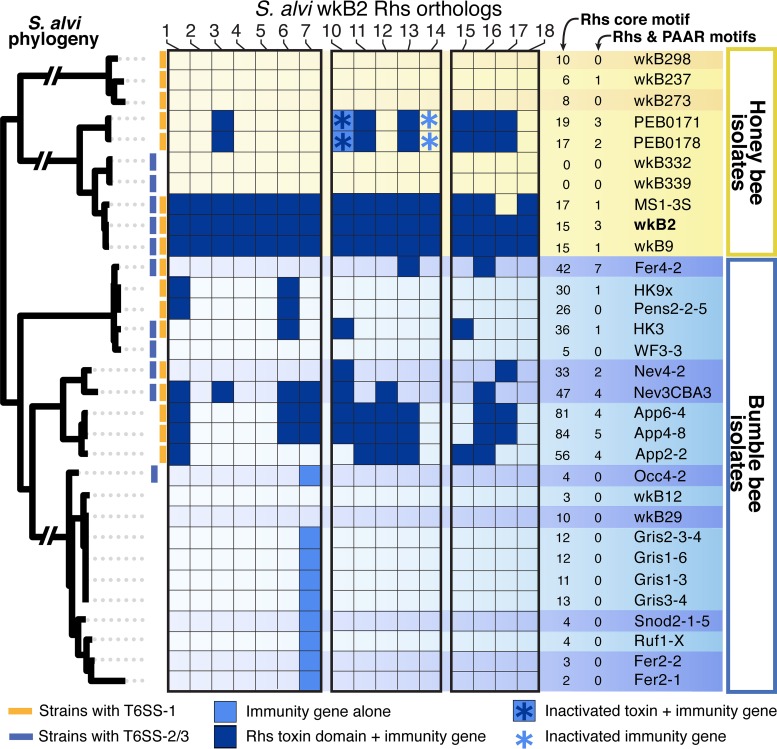
Rhs toxins and cognate immunity genes cooccur and are tightly linked. The presence/absence of *S. alvi* wkB2 C-terminal toxin domains and their cognate immunity gene in other *S. alvi* strains is shown. *rhs8* and *rhs9* lack toxin domains and immunity genes and are excluded. *S. alvi* strains are grouped according to strain phylogeny (left).

Frequent genetic transfers can break apart beneficial gene combinations, which would be highly detrimental in the case of a toxin and its immunity gene. The localization of Rhs immunity genes immediately adjacent to the C-terminal toxin domain is likely a mechanism that ensures against self-poisoning. We examine the linkage of Rhs toxin and immunity genes in *S. alvi* by searching the genomes of 20 *S. alvi* strains harboring T6SSs for the presence of *S. alvi* wkB2 toxins and their cognate immunity genes (Fig. 8). With the exception of isolates from Asian *Apis* species, every strain encoding T6SS-1 had at least one sequence homologous to a wkB2 toxin domain, and each of these also contained the associated immunity gene. In a few strains, immunity genes were detected without their associated toxin; however, the reverse was never true. The genomic context of these orphaned immunity genes suggests that they were originally acquired alongside their cognate toxin that has since been pseudogenized. Pseudogenization may also be the eventual fate of orphaned immunity genes in *S. alvi*, as several strains contain homologs to immunity genes from *S. alvi* wkB2 that have acquired nonsense mutations in the absence of the cognate toxin. Altogether, this illustrates both the importance of the immunity gene for the fitness of cells encoding the cognate Rhs toxin and the specificity of the toxin-immunity gene interaction.

### Rhs toxins undergo slow sequence evolution but rapid recombination.

Although coevolving systems often impose strong selection upon particular genes, homologous Rhs toxins have very little sequence variation, suggesting that rapid *de novo* mutation is not a major source of toxin diversity in the bee gut microbiota. We tested Rhs core and toxin domains for positive selection and found that both regions were under purifying selection (ratio of nonsynonymous to synonymous changes per nucleotide site [*dN/dS*] of <1), albeit with more relaxed constraints relative to highly conserved housekeeping genes (Fig. S4; Table S3).

10.1128/mBio.01630-17.4 FIG S4  Rhs toxins are under purifying selection and affect *S. alvi* fitness. (A) *dN/dS* ratios for Rhs C-terminal toxin domains were not significantly different from those of the Rhs core. Both Rhs domains had *dN/dS* ratios of <1 but significantly higher *dN/dS* ratios than conserved housekeeping genes (one-way ANOVA with Tukey’s multiple comparisons of means, *P*_adj_ < 0.005). Several homologous toxin domains were excluded from this analysis due to insufficient variation (i.e., only 0 to 5 polymorphisms). (B) Fold change in abundance of *S. alvi* wkB2 mutants with transposon insertions in Rhs toxins (top) and putative immunity genes (bottom) *in vivo*, relative to abundance *in vitro* (*P*_adj_ < 0.05). E, gene essential *in vitro*; NS, nonsignificant change in mutant abundance *in vivo*, **−**, immunity gene not present in genome. Gray arrows show the 5′-to-3′ organization of genes for each locus. Download FIG S4, PDF file, 0.2 MB. Copyright © 2017 Steele et al. 2017 Steele et al. This content is distributed under the terms of the Creative Commons Attribution 4.0 International license.

**FIG 9:**
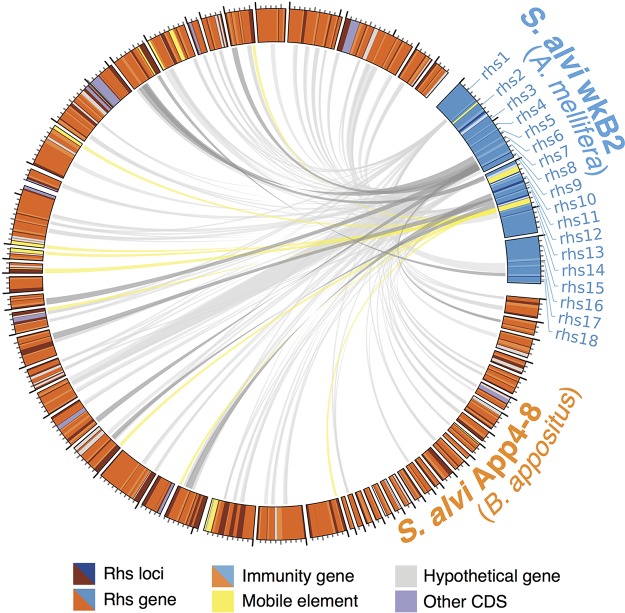
Rhs loci in divergent *S. alvi* strains share similar sequences. Dark and light gray ribbons show regions of >95% and >90% nucleotide similarity between *S. alvi* strains wkB2 and App4-8, respectively. Nine sequences within App4-8 Rhs loci have high identity (>95%) to 7 of the 18 Rhs genes in wkB2. Sequences similar to a putative integrase in wkB2 (yellow) are present in many App4-8 contigs. CDS, coding sequences.

As *de novo* mutation is not the primary source of Rhs diversity between strains, it is likely that the horizontal acquisition of novel toxin alleles and functional diversification through toxin/core recombination provide the adaptive basis of T6SS/Rhs-mediated competition in the bee gut microbiota. Comparison of the 3 Rhs loci in *S. alvi* wkB2 and the 52 Rhs-encoding contigs in *S. alvi* App4-8 identified several shared Rhs genes and a similar integrase-like gene in proximity to Rhs genes in the two genomes (Fig. 9). This gene, at >80% amino acid identity, was found in 19 *S. alvi* and 11 *G. apicola* genomes, while another Rhs-associated integrase was detected in 13 and 22 genomes of *S. alvi* and *G. apicola*, respectively. Such genes might facilitate the transfer and recombinatorial capture of Rhs sequences across gut microbiota strains, but further work will be necessary to determine whether these integrases contribute to the transfer of toxin genes between bee gut microbes.

## DISCUSSION

T6SS-mediated competition is likely to be an integral component of the ecology of *S. alvi* in the bee gut. Two phylogenetically distinct T6SSs were upregulated by *S. alvi* wkB2 in gnotobiotic *A. mellifera* workers. We determined that the toxins associated with one of these systems, T6SS-1, have antibacterial properties, which is consistent with a role in intraspecific competition. *S. alvi* strains with T6SS-1 can carry dozens of Rhs genes; some *Bombus*-derived strains have more than 100. We discovered a correspondingly large pool of toxins in the bee “pan-microbiome,” with over 1,000 Rhs genes and up to 364 different toxin domains detected across *S. alvi* and *G. apicola* strains isolated from *Apis* and *Bombus* hosts.

Given these results, we were surprised to find that many *S. alvi* and *G. apicola* strains completely lacked T6SSs and/or Rhs genes (Fig. 2A and 7). It is possible that these strains utilize other toxins or mechanisms of intercellular antagonism that we have not yet identified. However, the absence of these genes may be due to fitness tradeoffs involved in maintaining large, energy-intensive T6SSs and toxins that can cause self-poisoning. In fact, inactivation of T6SS and Rhs toxin genes by transposon insertion generally increases fitness *in vivo* in an *S. alvi* monospecies community (Fig. 5B). This suggests that, under some circumstances, loss of expensive competitive machinery in favor of maximizing growth may be a viable route for success within the gut community.

The two T6SSs found in *S. alvi* are strictly vertically inherited, meaning that loss of one or both systems is likely to be permanent. Accordingly, few *S. alvi* strains contain partial T6SSs, indicating that once a T6SS becomes nonfunctional, the remaining genes are rapidly deleted. In one large clade of *Bombus*-specific *S. alvi*, both T6SSs appear to be permanently lost, with a few strains retaining only the ends of the T6SS-2 locus (Fig. 2A and S1). This stands in sharp contrast with T6SS evolution in bacteria such as *Bacteroides* and *Salmonella*, where horizontal gene transfers of entire systems, possibly via association with mobile genetic elements, appear to be common (8, 11, 30).

Neither *S. alvi* T6SS-1 nor *S. alvi* T6SS-2/3 is closely related to T6SSs known to participate in virulence (Fig. 2B). Instead, *S. alvi* T6SS-1 clusters with the prokaryote-targeting HSI-I of *P. aeruginosa* PAO1 (31) and three *Burkholderia thailandensis* T6SSs, two of which are encoded within loci containing Rhs genes (32). The diverse sets of Rhs toxin genes associated with T6SS-1 suggest that it may have an important role in mediating intraspecific competition in *S. alvi*. However, this does not eliminate the possibility that T6SS-1 is also used to antagonize other Gram-negative bee gut microbes, including *G. apicola*, or even opportunistic pathogens. Recent studies have found that the bee gut microbiota reduces colonization by some opportunistic bacterial pathogens (33, 34), which makes it tempting to speculate that the *S. alvi* T6SSs may play a role in colonization resistance in the bee gut. We have demonstrated that Rhs toxins from *S. alvi* wkB2 have an antibacterial function (Fig. 6), but this does not eliminate the possibility that these toxins may also be able to antagonize eukaryotic cells, as T6SS effectors in other systems are toxic to both prokaryotes and eukaryotes (2, 29). *S. alvi* T6SS-2/3 is more closely related to those of bee-associated gammaproteobacteria, including *G. apicola* and *F. perrara*. *F. perrara* is part of the core honey bee gut microbiota but appears to cause damage to host tissue, triggering melanization at the site of colonization (35). The function of *S. alvi* T6SS-2/3 is not yet known. It is likely to be antibacterial, like T6SS-1, but T6SSs can also be used for host interaction. For instance, T6SS effectors are used by *Vibrio cholerae* to kill eukaryotic cells (1), and *Yersinia pestis* requires a T6SS to enter and grow inside human macrophages (36). However, this T6SS is unlikely to be used in virulence, as *S. alvi* is a commensal member of the bee gut microbiota and is not known to harm its host.

In contrast to the vertically inherited T6SSs in *S. alvi*, we find evidence that Rhs toxins may be horizontally transferred between strains. Strains from different hosts can share the same toxins, and some toxins are found within both *S. alvi* and *G. apicola*. *S. alvi* harbors a greater diversity and abundance of Rhs genes than *G. apicola*, suggesting that *S. alvi* may be the predominant reservoir for these toxins in the bee gut community. Additionally, the Rhs-associated T6SS-1 system was not found in *G. apicola*, which may constrain the types of toxins that it can secrete. There is evidence that T6SS-associated toxins are shared among strains and species in other systems (37–40). Furthermore, the potential for gene exchange in the bee gut is supported by our finding of near-identical mobile elements in divergent bacteria (Fig. 9), as well as previous evidence from whole-genome analyses (17) and the presence of identical antibiotic resistance determinants in *G. apicola* and *S. alvi* (41).

Further sampling will be necessary to explain the observed toxin distribution pattern (Fig. 7), as it is not yet clear what factors allow for the exchange of toxin genes between strains. Geographic isolation could explain the small and nonoverlapping assortment of Rhs genes found in honey bees from Southeast Asia compared to North American samples; however, this is confounded by the fact that these Asian bees represent different *Apis* spp. and come from a region where *Bombus* does not occur. That North American *S. alvi* strains from *A. mellifera* and *Bombus* species encode similar toxins is also intriguing, as *S. alvi* strains are host specialized, and the same strains do not typically reside in both *Apis* and *Bombus* hosts (13, 17, 19). Most toxins are not shared between *G. apicola* and *S. alvi*, which is consistent with observations that some Rhs genes are primarily used for competition between closely related strains or species (7, 27). Barriers to cross-species toxin exchange may explain these patterns but do not eliminate the possibility of Rhs-mediated interspecies competition. Rhs toxins potentially influence the capability of strains or species to coexist in the same gut community, a competitive interaction that has been implied for other antibacterial effectors (8, 39, 42). Interestingly, *G. apicola* wkB1 and *S. alvi* wkB2, which were isolated from the same *A. mellifera* colony at the same time, share a large proportion of Rhs genes—11 toxins and 12 immunity genes (17).

Many coevolving systems impose strong selection on traits that enhance competitiveness, leading to ongoing evolutionary responses in interacting lineages. The two major types of antagonistic coevolution are fluctuating selection dynamics and arms race dynamics (43). Evolutionary arms races typically feature rapid sequence evolution of proteins mediating the interaction (43, 44). However, we observed that very similar Rhs toxins are shared among distantly related bacterial strains. Rhs evolution also appears to be dominated by purifying selection (*dN/dS* ratio of <1), suggesting that positive selection for amino acid replacements is not a major mode of toxin evolution in these genes. These observations weigh against the possibility that Rhs evolution is driven by an evolutionary arms race. Instead, our results are more congruent with a fluctuating selection dynamics model of coevolution, which involves frequency-dependent selection favoring rare types (43). Phylogenetic studies suggest that *S. alvi* has evolved within bee hosts for over 80 million years (14); the large pool of Rhs toxins that has accumulated over this time may reflect the ever-changing competitive dynamics at play in bee gut communities. Frequency-dependent selection may prevent rare toxins from being lost, while the metabolic expense of maintaining T6SSs and toxins is likely to constrain the diversity of competitive machinery encoded by a single cell, ensuring that fluctuating selection dynamics remain predominant (44).

As in several enterobacterial species (45), the Rhs loci of *S. alvi* are comprised of a large, complete Rhs toxin gene at the 5′ end, followed by its cognate immunity gene, and then a series of truncated Rhs genes, or “orphaned” toxin domains, along with their immunity genes. This genetic architecture likely arises from “C-terminal displacement,” the exchange of toxin domains through recombination between conserved core regions (45, 46), which can generate functional diversity by packaging novel combinations of C-terminal toxin domains and N-terminal secretion domains. This mechanism also results in the genomic accrual of remnant, presumably nonsecreted Rhs toxin domains, which can subsequently be rerecruited into action via recombination (45, 46). In contrast to *Serratia* (45), the orphaned toxin domains in *S. alvi* wkB2 are not all silenced by nonsense mutations or missing translational start sites. Instead, many of these genes are transcribed and appear to produce proteins with antibacterial function, as inhibition of growth is observed when these toxins are heterologously expressed in *E. coli* (Fig. 6 and S3), and inactivating transposon insertions in their cognate immunity genes are detrimental to the fitness of *S. alvi* (Fig. 5B). Expression of *S. alvi* toxin and immunity genes in *E. coli* reveals that the protective effect of each immunity gene is specific to its cognate toxin, which is consistent with what has been observed in other species (27, 47). We also observed a correlation between the gene expression and gene position in two of the three Rhs loci in *S. alvi* wkB2, with the highest expression at the 5′ end and the lowest expression at the 3′ end, consistent with the direction of transcription and probable promoter placement.

The accumulation of numerous, potentially functional Rhs genes in *S. alvi* stands in contrast to what is found in many previously characterized bacteria: *Serratia marcescens* has two Rhs toxins (27), *Dickeya dadantii* 3937 has five (47), and *E. coli* K-12 has eight, including orphans (45). The conditions that promote the toxin expansion seen in *S. alvi* are unclear. Possibly, specialized bacteria living in communities with fewer species or more constrained niches might experience greater pressure to acquire diverse toxin and immunity gene pairs to compete against close relatives. However, this does not seem to be universally true of host-associated bacteria, as a recent survey of T6SS effectors in human gut *Bacteroidales* identified relatively few toxin and immunity gene pairs within individual strains and metagenomes (10). Though single strains of *Bacteroides fragilis* dominate the human gut microbiome, multiple *S. alvi* strains coexist within the bee gut (13), and interactions between these strains may contribute to increased toxin diversification in *S. alvi*. Additionally, the organization of microbes within individual bees or across individuals within the hive may allow for maintenance of otherwise incompatible sets of toxin and immunity genes, ultimately allowing for greater toxin diversity. It would be interesting to determine whether the diversity of Rhs toxins or other effectors correlates with host characteristics or gut community diversity or variability. Particularly, it is curious why *S. alvi* and *G. apicola* strains from *Bombus* hosts tend to encode far more Rhs toxins than do their counterparts in *Apis* species. In contrast to *Apis* species, *Bombus* species—and their gut microbiota—go through an annual population bottleneck when individual queens found new colonies, resulting in lower *S. alvi* strain diversity per individual bee (13). Potentially, the *Bombus* life cycle may impose more random fluctuations in the intensity of T6SS-mediated competition within their gut communities. Such differences in host ecology may help to explain why strains associated with some host species have accumulated large numbers of toxin genes while strains from other hosts have lost T6SSs entirely.

### Conclusion.

This study broadens our knowledge of the diversity of T6SSs and their effectors and highlights their potential role in shaping host-associated microbiomes. While the T6SS has been well studied in the decade since its discovery, the evolution and ecological role of these systems in naturally occurring polymicrobial communities have received relatively little attention until recently (48). We found that *S. alvi*, a resident member of the bee gut microbiota, encodes two T6SSs as well as numerous Rhs toxins, which are expressed *in vivo* and have antibacterial activities. These T6SSs were maintained during the diversification of *S. alvi* strains, suggesting that intercellular competition is important in the gut communities of diverse *Apis* and *Bombus* species. An enormous diversity of toxins was identified across the genomes of *S. alvi* and *G. apicola*, another bee gut resident. These toxins were often shared between distantly related strains, as well as between these two species, which represent different classes of *Proteobacteria*.

However, we also found that T6SS presence and toxin abundance vary among strains. The loss of T6SSs in some lineages indicates that participation in this mode of competition may not always be beneficial. Human gut bacteria with and without T6SSs have been shown to coexist in gnotobiotic mouse models (8). Clearly, T6SSs are only one component of bacterial competitiveness in gut communities. Other factors, such as metabolic tradeoffs, spatial distribution, and coevolutionary dynamics, will need to be considered to better understand the influence of T6SSs and associated effectors on microbial community structure, particularly over longer timescales. Furthermore, the bee gut microbiota has recently been shown to affect resistance to opportunistic bacterial pathogens that invade the body cavity through the gut (33, 34). Potentially, the T6SSs and Rhs toxins of *S. alvi* and *G. apicola* contribute to resistance to invasion by potential pathogens, such as strains of *Serratia* and other *Enterobacteriaceae*. The specialized gut microbiota of social bees presents a promising system in which to investigate these questions and will undoubtedly offer further insights into competition within coevolving bacterial communities.

## MATERIALS AND METHODS

### RNA sequencing.

For the *in vitro* samples, *S. alvi* wkB2 (49) was streaked in triplicate on heart infusion agar plates supplemented with 5% defibrinated sheep’s blood (HIA plus 5% SB) and incubated at 35°C in a 5% CO_2_ environment for 24 h. RNA was extracted from plated cells using TRIzol (Ambion), according to the manufacturer’s instructions.

For *in vivo* samples, microbiota-free bees were acquired as previously described (50). Briefly, pupae were extracted from brood frames of managed hives in New Haven, CT (experiment A), and Austin, TX (experiment B), and allowed to emerge under sterile conditions. Within 36 h of emergence, bees were fed 5 µl of a 20% sucrose–phosphate-buffered saline (PBS) solution containing approximately 10^6^ *S. alvi* wkB2 cells (optical density at 600 nm [OD_600_] of 0.5) or sterile PBS. Inoculated bees were transferred to sterile cup cages in triplicate groups of 8 to 10 and fed a filter-sterilized 1:1 sucrose solution and gamma-irradiated pollen *ad libitum*.

Four (experiment A) or 5 (experiment B) days after inoculation, bees were frozen at −80°C and then dissected on ice. Guts were placed in RNAlater (Thermo Fisher) and stored at −80°C. In experiment A, each replicate was comprised of three pooled ileums, while replicates in experiment B consisted of ileums and rectums of individual bees. For experiment B, DNA and RNA were extracted using the bead-beating and RNA-Bee method described by Jorth et al. (51). The absolute numbers of *S. alvi* 16S rRNA (RNA) and rRNA gene (DNA) copies in experiment B samples were quantified in triplicate with a 10^9^- to 10^3^-copy standard curve using the Beta-1009-qtF, Beta-1115-qtR, Gamma1-459-qtF, and Gamma1-648-qtR primers described in reference 18 on an Eppendorf Mastercycler EP RealPlex as described in reference 50. The Thermo Scientific Verso cDNA kit was used to synthesize cDNA for quantitative PCR (qPCR) according to the manufacturer’s instructions. Eukaryotic and prokaryotic rRNA was depleted using the Ribo-Zero Gold rRNA removal kit (Epidemiology).

The University of Texas at Austin Genomic Sequencing and Analysis Facility prepared stranded Illumina libraries for *in vitro* and experiment B samples and performed single-end 50-bp sequencing with an Illumina HiSeq4000 sequencer. Extraction and sequencing for experiment A are described in reference 23.

### Read processing and analysis.

RNA-Seq reads were trimmed with Flexbar (52) to remove Illumina adapters, and Bowtie2 (53) was used to map trimmed reads to the *S. alvi* wkB2 genome. HTSeq-count (54) was used to count the number of reads mapping to each gene in the RAST annotation. Differential expression analysis was done with DESeq2 (55), using a false discovery rate cutoff of 0.05. Transcripts per million (TPM) were calculated using a custom R script based on the calculation described in reference 56. One experiment B replicate had reads that mapped to other bacteria and was excluded from the analysis. Pearson’s coefficients for the relationship between Rhs toxin gene TPM and position for each Rhs locus were calculated using the corr.test function implemented in R (57).

### Reanalysis of Tn-Seq data.

The T6SS and Rhs loci of the NCBI-annotated *S. alvi* wkB2 genome (CP007446.1) were reannotated by locating open reading frames (ORFs) and performing blastp searches of the predicted proteins. The reannotated genome was used as the reference on which Tn-Seq data from a previous study (23) were remapped. Scoring of essential genes and genes beneficial *in vivo* was performed as described previously (23).

### Genome sequencing.

DNA was extracted from *S. alvi* cultures using the Qiagen blood and tissue kit and submitted for library preparation and sequencing using the Illumina MiSeq platform with paired-end, 2- by 250-bp or 2- by 300-bp reads. Genomes were assembled with Velvet (58) or MaSuRCA (59) and annotated using the RAST (24) pipeline.

### Identification of T6SS loci and correlation between T6SS-1 and Rhs genes.

T6SS homologs were identified by amino acid similarity to the T6SS-associated genes of *S. alvi* wkB2 using tblastx best hits with an E value cutoff of ≤10^−5^, coverage of ≥50%, and ≥50% identity. A 70% identity cutoff was used for *tssH*. tblastx (E value, <0.01) was used to determine the average sequence similarity of the proteins encoded by the T6SS-1 and T6SS-2/3 loci of wkB2. Four genes (*tssA*, *tssE*, *tssJ*, and *tssM*) had no matches below this threshold. For the nine remaining core T6SS genes, percent identity for each match was normalized by the percentage of residues in the query sequence that aligned to the reference sequence, and the average of these values was taken. Open reading frame (ORF) maps for wkB2 T6SS loci were visualized using Geneious 10.1.3. HMMER 3.0 (60) was used to identify *S. alvi* protein coding genes containing the Rhs core motif (TIGR03696) and to determine the number of these proteins that also contain a PAAR motif (PF05488). Strains were then grouped based on the presence of T6SS-1, T6SS-2/3, both, or neither. Rhs gene counts were compared between groups using a one-way analysis of variance (ANOVA) with Tukey’s honestly significant difference (HSD) multiple-test correction.

### Cloning Rhs toxin and immunity genes.

Standard restriction enzyme cloning methods were used to clone *rhs1* and *rhs2* into pET21a and *rhs2I* into pJN105, whereas Gibson assembly (61) was used to clone *rhs17* into pET21a and *rhs1I* and *rhs17I* into pJN105. Genes were amplified with Phusion DNA polymerase (New England Biolabs) and the primers listed in Table S6 in the supplemental material. Digestions were performed using NdeI and XhoI (*rhs1*) or NdeI and NotI (*rhs2*), and T4 DNA ligase (New England Biolabs) was used to catalyze ligations. The *rhs1* forward primer was designed to generate a truncated C-terminal toxin domain comprised of the last 146 amino acids of the toxin. Electroporation was used to transform ligation and Gibson assembly reaction mixtures into *E. coli* DH5α, and PCR was used to screen transformants for the presence of the correct insert. Inserts were verified by sequencing the cloning site of the purified plasmid. All insert sequences matched the *S. alvi* wkB2 genome exactly, except for *rhs2*, which has a nonsense mutation 99 nucleotides before the end of the gene.

10.1128/mBio.01630-17.10 TABLE S6  Primers used to clone Rhs toxin and immunity genes into expression vectors. Download TABLE S6, DOCX file, 0.1 MB. Copyright © 2017 Steele et al. 2017 Steele et al. This content is distributed under the terms of the Creative Commons Attribution 4.0 International license.

### Expression of Rhs toxin and immunity genes in *E. coli.*

Purified plasmids were transformed into *E. coli* BL21(DE3) via electroporation for induction assays. Cells containing either pET21a::toxin and pJN105::immunity, pET21a::toxin and pJN105 empty vector, or pJN105::immunity and pET21a empty vector were streaked out on LB plates containing 10 µg/ml gentamicin, 75 µg/ml ampicillin, and 1% glucose and incubated overnight at 37°C. Single colonies were picked from these plates and streaked out onto fresh 10-µg/ml gentamicin and 75-µg/ml ampicillin plates with and without 0.1 mM IPTG or 0.5% l-arabinose. Growth was observed after 24 h at 37°C.

Growth curves were constructed to measure the effect of toxin and immunity gene induction on growth over time. Cells containing pET21a with or without a toxin gene and pJN105 with or without the immunity gene were streaked out on LB plates containing 10 µg/ml gentamicin and 75 µg/ml ampicillin and incubated overnight. Single colonies were used to inoculate 4 ml LB with selection in a 14-ml culture tube, and these cultures were grown to mid-log phase at 37°C and 220 rpm, then diluted back to an OD_600_ of 0.01 and incubated for another 3 h, and then diluted to an OD_600_ of 0.2. Ten microliters of this culture was added to 190 µl LB broth with selection, with and without IPTG or l-arabinose, in a 96-well plate. A Tecan Spark 10 M plate reader was used to measure the OD_600_ of cultures every 30 min for 12 h.

### Phylogenetic trees.

Concatenated nucleotide sequences of 37 ribosomal protein genes (Table S3) were used to construct a phylogenetic tree for 31 *S. alvi* strains. Sequences were aligned with MUSCLE 3.8.31 (62). Bayesian and maximum likelihood (ML) analyses were performed using MrBayes 3.2 (63) and RAxML v8 (64), respectively. Both Bayesian and ML analyses used a general time-reversible model of nucleotide substitution with a proportion of sites assumed to be invariable and the remaining sites drawn from a gamma distribution. The Bayesian analysis was run until the standard deviation of split frequencies dropped below 0.01. Bootstrapping was performed for the ML analysis (*n* = 1,000). Phylogenetic trees were visualized with FigTree v.1.4.2 (65).

Phylogenetic trees of *S. alvi* T6SS-1 and T6SS-2/3 were constructed using the same method. T6SS homologs were extracted from a local BLAST database of *S. alvi* protein-encoding genes based on amino acid similarity to T6SS-associated genes in wkB2. The only gene from the wkB2 T6SS-1 locus that had amino acid identity to its T6SS-2/3 homolog above our cutoff was *tssC*.

The TssB protein is well conserved and has previously been used to determine evolutionary relationships between distantly related T6SSs (32, 66). A phylogeny of the TssB protein was constructed using representative sequences from *S. alvi* and *G. apicola*, as well as reference sequences from the NCBI RefSeq database. The SecReT6 database (67) was used to identify proteins associated with previously identified T6SS subtypes. CD-HIT (68) was used to cluster *S. alvi* and *G. apicola* TssB sequences by 95% similarity, and MUSCLE was used to align representative sequences from each cluster with the reference sequences. Maximum likelihood analyses were performed with RAxML v8 using the WAG amino acid substitution model and assuming a gamma distribution of rates with a proportion of invariable sites and 1,000 bootstrap replicates.

### Comparison of Rhs loci.

Conserved domains in the *S. alvi* wkB2 Rhs genes were identified using the NCBI’s conserved domain database (28) with a 10^−5^ E value cutoff. C-terminal toxin domains were identified based on the location of the conserved DPXG(18)DPXG motif in the translated sequence. Homologous toxin domains were detected by comparing translated sequences of the wkB2 C-terminal domains to all *S. alvi* genomes with the tblastx (69) tool and 70% coverage and 70% amino acid identity cutoffs.

Rhs loci were identified using RAST annotations and manually edited in Geneious R9 (70). Regions of high primary sequence homology between *S. alvi* Rhs loci were identified using blastn (69) with a 10^−5^ E value cutoff and visualized with Circos (71).

To obtain the Rhs toxin cooccurrence network, C-terminal toxin domains were extracted from a codon alignment of all *S. alvi* and *G. apicola* Rhs genes and clustered at 90% nucleotide identity with CD-HIT-est (56). Predicted C-terminal domains shorter than 40 bp were excluded. From this, 364 unique toxin domains were identified. A more conservative analysis, in which a more stringent match requirement for the DPXG motif and a 200-bp length cutoff were applied, revealed 258 unique C-terminal domains. Output was visualized in Gephi v0.9.1 (72).

### Tests of selection.

Homologous conserved gene and Rhs sequences (Table S3) were identified in *S. alvi* genomes using tblastx, based on amino acid identity to *S. alvi* wkB2 genes. Alignments were constructed with MUSCLE and manually checked in Geneious R9. Omega (*dN/dS*) ratios were calculated using the PAML 4.8 codeml model 0 (73). A one-way ANOVA with Tukey’s HSD multiple-test correction was used to compare the *dN/dS* ratios of conserved genes, Rhs core domains, and Rhs toxin domains.

### Accession number(s).

RNA-Seq data from experiment B are deposited under accession number SRP082731 in the NCBI Sequence Read Archive. Genome sequences are deposited under accession numbers MDUY00000000, MDUZ00000000, MDVA00000000, MDVB00000000, MDVC00000000, MDVD00000000, MDVE00000000, MDVF00000000, MDVG00000000, MDVH00000000, MDVI00000000, MDVJ00000000, MEII00000000, MEIJ00000000, MEIK00000000, MEIL00000000, MEIM00000000, MEIN00000000, MEIO00000000, MEIP00000000, MEIQ00000000, MEIR00000000, MEIS00000000, MEIT00000000, MEIU00000000, MEIV00000000, MEIW00000000, and MEIX00000000 in GenBank.
