# Supplementary material for: Diversification of Type VI Secretion System Toxins Reveals Ancient Antagonism among Bee Gut Microbes
Source: mBio. 2017 Dec 12;8(6):e01630-17. doi: 10.1128/mBio.01630-17 (PMC5727410; doi:10.1128/mBio.01630-17)
Supplement: TABLE S1 [file mbo006173631st1.docx]

**Table S1.** Differential expression of *S. alvi* wkB2 T6SS genes, Rhs toxin and putative immunity genes, and genes involved in iron and nitrogen metabolism.

|  |  |  |  | **Experiment A** | | **Experiment B** | |  |  |  |  |
| --- | --- | --- | --- | --- | --- | --- | --- | --- | --- | --- | --- |
| **Category** | **Gene ID** | **Gene** | **Locus** | **log_2_ fold change** | **p_adj_** | **log_2_ fold change** | **p_adj_** | **Significant in** | **Direction** | **COG** | **KEGG** |
| T6SS | SALWKB2_0097 | *tssA* | T6SS-1 | 1.031 | 2.89E-06 | 1.345 | 1.61E-07 | Exp. A & B | Upregulated | COG3515 | K11902 |
| T6SS | SALWKB2_0098 | *tssG* | T6SS-1 | 1.434 | 1.40E-07 | 1.830 | 2.43E-08 | Exp. A & B | Upregulated | COG3520 | K11895 |
| T6SS | SALWKB2_0099 | *tssF* | T6SS-1 | 1.054 | 0.0001 | 1.578 | 5.30E-08 | Exp. A & B | Upregulated | COG3519 | K11896 |
| T6SS | SALWKB2_0100 | *tssE* | T6SS-1 | 0.998 | 9.78E-06 | 1.209 | 1.33E-05 | Exp. A & B | Upregulated | COG3518 | K11897 |
| T6SS | SALWKB2_0101 | hyp | T6SS-1 | 0.705 | 0.0386 | 0.555 | 0.1521 | Exp. A | Upregulated | COG4455 | K11898 |
| T6SS | SALWKB2_0102 | hyp | T6SS-1 | 1.252 | 3.09E-07 | 1.045 | 0.0003 | Exp. A & B | Upregulated |  |  |
| T6SS | SALWKB2_0103 | *tssH* | T6SS-1 | 1.196 | 0.0002 | 1.103 | 0.0021 | Exp. A & B | Upregulated | COG0542 | K11907 |
| T6SS | SALWKB2_0104 | *tssB* | T6SS-1 | 0.806 | 0.2875 | -0.143 | 0.8660 | NS |  | COG3516 | K11901 |
| T6SS | SALWKB2_0105 | *tssC* | T6SS-1 | 1.155 | 0.0930 | 0.048 | 0.9503 | NS |  | COG3517 | K11900 |
| T6SS | SALWKB2_0106 | *tssD* | T6SS-1 | 1.086 | 0.2329 | -0.429 | 0.6608 | NS |  | COG3157 | K11903 |
| T6SS | SALWKB2_0107 | *tssJ* | T6SS-1 | 1.635 | 1.02E-09 | 1.150 | 0.0003 | Exp. A & B | Upregulated | COG3521 | K11906 |
| T6SS | SALWKB2_0108 | *tssK* | T6SS-1 | 1.436 | 1.17E-07 | 1.372 | 6.67E-06 | Exp. A & B | Upregulated | COG3522 | K11893 |
| T6SS | SALWKB2_0109 | *tssL* | T6SS-1 | 0.963 | 0.0006 | 0.912 | 0.0042 | Exp. A & B | Upregulated | COG1360 | K11892 |
| T6SS | SALWKB2_0110 | hyp | T6SS-1 | 0.668 | 0.0617 | 0.020 | 0.9708 | NS |  |  |  |
| T6SS | SALWKB2_0111 | hyp | T6SS-1 | 0.761 | 0.0053 | 0.259 | 0.5022 | Exp. A | Upregulated |  |  |
| T6SS | SALWKB2_0112 | *tssM* | T6SS-1 | 1.253 | 8.40E-06 | 1.239 | 0.0001 | Exp. A & B | Upregulated | COG3523 | K11891 |
| T6SS | SALWKB2_0113 | hyp | T6SS-1 | 1.174 | 4.55E-05 | 1.197 | 0.0005 | Exp. A & B | Upregulated | COG3913 |  |
| T6SS | SALWKB2_0114 | hyp | T6SS-1 | 1.979 | 6.36E-06 | 1.691 | 0.0041 | Exp. A & B | Upregulated |  |  |
| T6SS | SALWKB2_0116 | *tssI* | T6SS-1 | 1.625 | 1.01E-12 | 2.061 | 3.89E-16 | Exp. A & B | Upregulated | COG3501 | K11904 |
| T6SS | SALWKB2_0913 | *tssI* | T6SS-2 | 1.439 | 4.72E-11 | 1.121 | 7.68E-06 | Exp. A & B | Upregulated | COG4253 | K11904 |
| T6SS | SALWKB2_0914 | hyp | T6SS-2 | 0.884 | 0.0002 | 0.271 | 0.4011 | Exp. A | Upregulated |  |  |
| T6SS | SALWKB2_0915 | hyp | T6SS-2 | 0.616 | 0.0009 | -0.203 | 0.4513 | Exp. A | Upregulated | COG0790 | K07126 |
| T6SS | SALWKB2_0916 | hyp | T6SS-2 | 1.092 | 4.32E-06 | -0.044 | 0.9059 | Exp. A | Upregulated |  |  |
| T6SS | SALWKB2_0917 | *tssM* | T6SS-2 | 1.181 | 1.51E-06 | 0.482 | 0.1118 | Exp. A | Upregulated | COG3523 | K11891 |
| T6SS | SALWKB2_0918 | *tssA* | T6SS-2 | 1.073 | 7.54E-09 | 0.642 | 0.0095 | Exp. A & B | Upregulated | COG3515 | K11910 |
| T6SS | SALWKB2_0919 | *tssF* | T6SS-2 | 1.244 | 4.97E-06 | 0.740 | 0.0263 | Exp. A & B | Upregulated | COG3519 | K11896 |
| T6SS | SALWKB2_0920 | *tssG* | T6SS-2 | 1.184 | 0.0003 | 0.365 | 0.4184 | Exp. A | Upregulated | COG3520 | K11895 |
| T6SS | SALWKB2_0921 | *tssJ* | T6SS-2 | 1.058 | 0.0130 | 1.097 | 0.0216 | Exp. A & B | Upregulated | COG3521 |  |
| T6SS | SALWKB2_0922 | *tssE* | T6SS-2 | 0.798 | 0.0171 | -0.090 | 0.8715 | Exp. A | Upregulated | COG3518 | K11905 |
| T6SS | SALWKB2_1360 | *tssH* | T6SS-3 | 0.951 | 1.54E-05 | 0.123 | 0.6748 | Exp. A | Upregulated | COG0542 | K11907 |
| T6SS | SALWKB2_1361 | *tssD* | T6SS-3 | 1.115 | 0.0021 | -0.792 | 0.0489 | Exp. A & B | Split | COG3157 | K11903 |
| T6SS | SALWKB2_1362 | *tssL* | T6SS-3 | 1.253 | 0.0001 | -0.658 | 0.0766 | Exp. A | Upregulated | COG2885 |  |
| T6SS | SALWKB2_1363 | *tssL* | T6SS-3 | 0.597 | 0.0602 | -0.435 | 0.2694 | NS |  | COG3455 |  |
| T6SS | SALWKB2_1364 | *tssK* | T6SS-3 | 1.033 | 0.0002 | 0.239 | 0.4968 | Exp. A | Upregulated | COG3522 | K11893 |
| T6SS | SALWKB2_1365 | hyp | T6SS-3 | 1.552 | 2.34E-11 | 0.747 | 0.0204 | Exp. A & B | Upregulated | COG4104 |  |
| T6SS | SALWKB2_1366 | *tssC* | T6SS-3 | 1.144 | 0.0010 | 0.496 | 0.2168 | Exp. A | Upregulated | COG3517 | K11900 |
| T6SS | SALWKB2_1367 | *tssB* | T6SS-3 | 0.562 | 0.2061 | -0.109 | 0.8339 | NS |  | COG3516 | K11901 |
| T6SS | SALWKB2_1932 | *tssD* | NA | 0.812 | 0.0220 | 2.768 | 2.78E-16 | Exp. A & B | Upregulated | COG3157 |  |
| **Category** | **Gene ID** | **Gene** | **Locus** | **log_2_ fold change** | **p_adj_** | **log_2_ fold change** | **p_adj_** | **Significant in** | **Direction** | **COG** | **KEGG** |
| Immunity | SALWKB2_0117 | *rhs1I* | Rhs-I | 0.621 | 0.0107 | -0.031 | 0.9310 | Exp. A | Upregulated | COG5435 |  |
| Toxin | SALWKB2_0118 | *rhs1* | Rhs-I | 1.170 | 2.54E-05 | 1.791 | 3.10E-09 | Exp. A & B | Upregulated | COG3209 |  |
| Toxin | SALWKB2_0120 | *rhs2* | Rhs-I | 0.636 | 0.0110 | 0.532 | 0.0643 | Exp. A | Upregulated | COG3209 |  |
| Immunity | SALWKB2_0121 | *rhs2I* | Rhs-I | 0.468 | 0.0845 | -0.102 | 0.7972 | NS |  |  |  |
| Pseudotoxin | SALWKB2_0124 | *rhs3* | Rhs-I | -0.029 | 0.9441 | 0.214 | 0.5755 | NS |  | COG3209 |  |
| Immunity | SALWKB2_0125 | *rhs3I* | Rhs-I | 0.307 | 0.6050 | 0.079 | 0.9195 | NS |  |  |  |
| Toxin | SALWKB2_0126 | *rhs4* | Rhs-I | 0.277 | 0.4162 | -0.357 | 0.3392 | NS |  | COG3209 |  |
| Immunity | SALWKB2_0126_5 | *rhs4I* | Rhs-I | -0.359 | 0.2893 | -0.839 | 0.0266 | Exp. B | Downregulated | |  |
| Toxin | SALWKB2_0127 | *rhs5* | Rhs-I | 0.641 | 0.0014 | 0.635 | 0.0097 | Exp. A & B | Upregulated | COG3209 |  |
| Immunity | SALWKB2_0128 | *rhs5I* | Rhs-I | 1.379 | 0.0001 | -0.198 | 0.7809 | Exp. A | Upregulated |  |  |
| Toxin | SALWKB2_0129 | *rhs6* | Rhs-I | 0.809 | 0.0010 | 0.374 | 0.2777 | Exp. A | Upregulated | COG3209 |  |
| Immunity | SALWKB2_0130 | *rhs6I* | Rhs-I | 0.919 | 0.0752 | 0.782 | 0.2680 | NS |  |  |  |
| Toxin | SALWKB2_0131 | *rhs7* | Rhs-I | 0.555 | 0.0420 | 1.261 | 2.89E-05 | Exp. A & B | Upregulated | COG3209 |  |
| Immunity | SALWKB2_0132 | *rhs7I* | Rhs-I | 0.024 | 0.9661 | 0.756 | 0.1631 | NS |  |  |  |
| Pseudotoxin | SALWKB2_0133 | *rhs8* | Rhs-I | 1.889 | 0.0053 | 3.786 | 9.83E-09 | Exp. A & B | Upregulated | COG3209 |  |
| Pseudotoxin | SALWKB2_1253 | *rhs9* | Rhs-II | 0.460 | NA | 2.763 | 0.0035 | Exp. B | Upregulated | COG3209 |  |
| Immunity | SALWKB2_1256_5 | *rhs10I* | Rhs-II | -0.174 | 0.7640 | 0.373 | 0.5441 | NS |  |  |  |
| Toxin | SALWKB2_1257 | *rhs10* | Rhs-II | 1.080 | 0.0066 | 2.040 | 2.76E-06 | Exp. A & B | Upregulated | COG3209 |  |
| Immunity | SALWKB2_1258 | *rhs11I* | Rhs-II | -0.114 | 0.8562 | 1.037 | 0.0524 | NS |  |  |  |
| Toxin | SALWKB2_1259 | *rhs11* | Rhs-II | 0.090 | 0.8344 | 0.889 | 0.0166 | Exp. B | Upregulated | COG3209 |  |
| Immunity | SALWKB2_1259_5 | *rhs12I* | Rhs-II | -1.134 | 0.0988 | -0.341 | 0.7030 | NS |  |  |  |
| Toxin | SALWKB2_1260 | *rhs12* | Rhs-II | 0.136 | 0.8703 | 2.128 | 0.0003 | Exp. B | Upregulated | COG3209 |  |
| Immunity | SALWKB2_1262 | *rhs13I* | Rhs-II | 0.212 | 0.3329 | -1.323 | 1.42E-05 | Exp. B | Downregulated | |  |
| Toxin | SALWKB2_1263 | *rhs13* | Rhs-II | 0.602 | 0.0026 | -0.350 | 0.2631 | Exp. A | Upregulated | COG3209 |  |
| Immunity | SALWKB2_1264 | *rhs14I* | Rhs-II | 0.585 | 0.0721 | -1.005 | 0.0412 | Exp. B | Downregulated | |  |
| Toxin | SALWKB2_1265 | *rhs14* | Rhs-II | 1.073 | 0.0002 | 1.644 | 2.12E-07 | Exp. A & B | Upregulated | COG3209 |  |
| Toxin | SALWKB2_2002 | *rhs15* | Rhs-III | 0.336 | 0.2947 | 1.921 | 3.68E-11 | Exp. B | Upregulated | COG3209 |  |
| Immunity | SALWKB2_2002_5 | *rhs15I* | Rhs-III | 0.366 | 0.1562 | -1.338 | 0.0031 | Exp. B | Downregulated | |  |
| Toxin | SALWKB2_2003 | *rhs16* | Rhs-III | 0.012 | 0.9657 | -0.331 | 0.2726 | NS |  | COG3209 |  |
| Immunity | SALWKB2_2004 | *rhs16I* | Rhs-III | 0.846 | 0.0294 | -0.321 | 0.6444 | Exp. A | Upregulated | COG3209 |  |
| Toxin | SALWKB2_2005 | *rhs17* | Rhs-III | 0.890 | 3.42E-05 | 1.137 | 3.17E-05 | Exp. A & B | Upregulated | COG3209 |  |
| Immunity | SALWKB2_2006 | *rhs17I* | Rhs-III | 0.712 | 0.0079 | -0.622 | 0.0677 | Exp. A | Upregulated | COG3209 |  |
| Toxin | SALWKB2_2007 | *rhs18* | Rhs-III | 0.835 | 1.80E-05 | 0.196 | 0.5047 | Exp. A | Upregulated | COG3209 |  |
| Immunity | SALWKB2_2007_5 | *rhs18I* | Rhs-III | -0.514 | 0.1147 | -2.022 | 0.0002 | Exp. B | Downregulated | |  |
| **Category** | **Gene ID** | **Gene** | **Locus** | **log_2_ fold change** | **p_adj_** | **log_2_ fold change** | **p_adj_** | **Significant in** | **Direction** | **COG** | **KEGG** |
| Iron metabolism | SALWKB2_0189 | *hmuS* |  | 2.144 | 3.66E-09 | 2.356 | 9.75E-09 | Exp. A & B | Upregulated | COG3720 | K07225 |
| Iron metabolism | SALWKB2_0190 | *fhuD* |  | 1.916 | 1.08E-09 | 3.563 | 6.44E-27 | Exp. A & B | Upregulated | COG4558 | K02016 |
| Iron metabolism | SALWKB2_0191 | *fhuB* |  | 1.499 | 3.81E-04 | 2.984 | 1.51E-11 | Exp. A & B | Upregulated | COG0609 | K02015 |
| Iron metabolism | SALWKB2_0192 | *fhuC* |  | 0.762 | 0.050 | 1.453 | 0.001 | Exp. A & B | Upregulated | COG1120 | K02013 |
| Iron metabolism | SALWKB2_1610 | *fhuD* |  | 0.808 | 0.023 | 1.227 | 0.001 | Exp. A & B | Upregulated | COG4607 | K02016 |
| Iron metabolism | SALWKB2_2008 | *entF* |  | 0.640 | 0.098 | 1.293 | 0.002 | Exp. B | Upregulated | COG1020 |  |
| Iron metabolism | SALWKB2_2009 | *entF* |  | 2.462 | 2.58E-07 | 1.542 | 0.006 | Exp. A & B | Upregulated | COG1020 |  |
| Iron metabolism | SALWKB2_2011 | *ceuB* |  | 1.087 | 0.027 | 0.214 | 0.737 | Exp. A | Upregulated | COG4606 | K02015 |
| Iron metabolism | SALWKB2_2012 | *ceuC* |  | 1.325 | 0.024 | 1.358 | 0.039 | Exp. A & B | Upregulated | COG4605 | K02015 |
| Iron metabolism | SALWKB2_2013 | *ceuD* |  | 1.458 | 0.011 | 1.443 | 0.024 | Exp. A & B | Upregulated | COG4604 | K02013 |
| Iron metabolism | SALWKB2_2014 | *ceuA* |  | 1.125 | 0.028 | -0.022 | 0.972 | Exp. A | Upregulated | COG4607 | K02016 |
| Iron metabolism | SALWKB2_2015 | *fiu* |  | 1.466 | 0.030 | 1.018 | 0.165 | Exp. A | Upregulated | COG4774 | K02014 |
| Iron metabolism | SALWKB2_2016 | *iucD* |  | 1.596 | 0.003 | 3.429 | 1.41E-10 | Exp. A & B | Upregulated | COG3486 | K03897 |
| Iron metabolism | SALWKB2_2017 | *entF* |  | 2.457 | 0.006 | 2.795 | 0.003 | Exp. A & B | Upregulated | COG1020 |  |
| Iron metabolism | SALWKB2_2018 | *entE* |  | 2.067 | 1.30E-07 | 2.180 | 7.92E-07 | Exp. A & B | Upregulated | COG1021 | K02363 |
| Iron metabolism | SALWKB2_2019 | *entB* |  | 1.774 | 1.51E-06 | 1.880 | 1.25E-05 | Exp. A & B | Upregulated | COG1535 | K01252 |
| Iron metabolism | SALWKB2_2020 | *mdlB* |  | 0.854 | 0.012 | 1.525 | 4.34E-05 | Exp. A & B | Upregulated | COG1132 |  |
| Iron metabolism | SALWKB2_2021 | *mdlB* |  | 0.929 | 0.031 | 1.286 | 0.006 | Exp. A & B | Upregulated | COG1132 |  |
| Iron metabolism | SALWKB2_2022 | *entD* |  | 1.420 | 0.003 | 3.166 | 2.80E-11 | Exp. A & B | Upregulated | COG2977 |  |
| Iron metabolism | SALWKB2_2023 | *entC* |  | 2.594 | 1.96E-06 | 2.982 | 8.99E-07 | Exp. A & B | Upregulated | COG1169 | K02361 |
| Iron metabolism | SALWKB2_2024 | *fabG* |  | 1.536 | 0.001 | 1.604 | 0.003 | Exp. A & B | Upregulated | COG1028 | K00216 |
| Nitrogen metab. | SALWKB2_0412 | *gltB1* |  | 0.237 | 0.308 | 0.370 | 0.110 | NS |  | COG0067 | K00265 |
| Nitrogen metab. | SALWKB2_0413 | *gltD* |  | 0.572 | 0.005 | 0.766 | 0.001 | Exp. A & B | Upregulated | COG0493 | K00266 |
| Nitrogen metab. | SALWKB2_0899 | *narK* |  | 1.072 | 0.040 | 2.317 | 2.22E-05 | Exp. A & B | Upregulated | COG2223 | K02575 |
| Nitrogen metab. | SALWKB2_0900 | *narK* |  | 1.502 | 8.46E-05 | 3.131 | 2.93E-15 | Exp. A & B | Upregulated | COG2223 | K02575 |
| Nitrogen metab. | SALWKB2_0901 | *narG* |  | 1.549 | 4.09E-07 | 2.925 | 4.10E-19 | Exp. A & B | Upregulated | COG5013 | K00370 |
| Nitrogen metab. | SALWKB2_0902 | *narH* |  | 0.942 | 0.003 | 1.618 | 3.71E-06 | Exp. A & B | Upregulated | COG1140 | K00371 |
| Nitrogen metab. | SALWKB2_0903 | *narJ* |  | 0.760 | 0.050 | 1.145 | 0.012 | Exp. A & B | Upregulated | COG2180 | K00373 |
| Nitrogen metab. | SALWKB2_0904 | *narI* |  | 0.034 | 0.954 | -0.043 | 0.942 | NS |  | COG2181 | K00374 |
| Nitrogen metab. | SALWKB2_2176 | *amtB* |  | 2.083 | 1.86E-08 | 2.178 | 1.31E-07 | Exp. A & B | Upregulated | COG0004 | K03320 |
| Nitrogen metab. | SALWKB2_2177 | *glnK* |  | 3.087 | 2.87E-13 | 2.457 | 2.91E-06 | Exp. A & B | Upregulated | COG0347 | K04751 |

hyp, hypothetical open reading frame; p_adj_, Benjamini-Hochberg adjusted p-values; NS, not significant.
