# Supplementary material for: Diversification of Type VI Secretion System Toxins Reveals Ancient Antagonism among Bee Gut Microbes
Source: mBio. 2017 Dec 12;8(6):e01630-17. doi: 10.1128/mBio.01630-17 (PMC5727410; doi:10.1128/mBio.01630-17)
Supplement: TABLE S2 [file mbo006173631st2.docx]

**Table S2.** *S. alvi* strains and genomes used in this study.

| **Strain** | **Host Species** | **Host Subgenus** | **# Contigs** | **Location** | **Collection date** | **Isolated by** | **­Accession # or reference** |
| --- | --- | --- | --- | --- | --- | --- | --- |
| wkB237 | *Apis andreniformis* | *Micrapis* | 21 | Hort Park, Singapore | 9-Aug-14 | W. Kwong | MEIM00000000 |
| wkB298 | *Apis cerana* | *Apis* | 42 | Hort Park, Singapore | 9-Aug-14 | W. Kwong | MEIK00000000 |
| wkB273 | *Apis florea* | *Micrapis* | 31 | Clementi Park, Singapore | 11-Aug-14 | W. Kwong | MEIL00000000 |
| wkB9 | *Apis mellifera* | *Apis* | 15 | New Haven, CT USA | 25-May-11 | W. Kwong | MEIN00000000 |
| PEB0171 | *Apis mellifera* | *Apis* | 78 | New Haven, CT USA | 10-Sep-11 | P. Engel | MEIV00000000 |
| wkB332 | *Apis mellifera* | *Apis* | 30 | Genting, Malaysia | 27-Jul-14 | W. Kwong | MEIJ00000000 |
| wkB339 | *Apis mellifera* | *Apis* | 27 | Genting, Malaysia | 27-Jul-14 | W. Kwong | MEII00000000 |
| PEB0178 | *Apis mellifera* | *Apis* | 136 | New Haven, CT USA | 10-Sep-11 | P. Engel | MEIW00000000 |
| wkB2 | *Apis mellifera* | *Apis* | 1 | New Haven, CT USA | 25-May-11 | W. Kwong | [11] |
| MS1-3S | *Apis mellifera* | *Apis* | 94 | Austin, TX USA | Feb-14 | M. Steele | MEIX00000000 |
| App2-2 | *Bombus appositus* | *Subterraneobombus* | 119 | Logan, UT USA | 7-Jul-2013 | H. Koch | MDVB00000000 |
| App4-8 | *Bombus appositus* | *Subterraneobombus* | 115 | Logan, UT USA | 7-Jul-2013 | H. Koch | MDVC00000000 |
| App6-4 | *Bombus appositus* | *Subterraneobombus* | 108 | Logan, UT USA | 7-Jul-2013 | H. Koch | MDVD00000000 |
| wkB12 | *Bombus bimaculatus* | *Pyrobombus* | 22 | New Haven, CT USA | 17-Jun-11 | W. Kwong | [11] |
| Fer1-2 | *Bombus fervidus* | *Thoracobombus* | 66 | Logan, UT USA | 8-Jul-2013 | H. Koch | MDVE00000000 |
| Fer2-2 | *Bombus fervidus* | *Thoracobombus* | 53 | Logan, UT USA | 8-Jul-2013 | H. Koch | MDVF00000000 |
| Fer4-2 | *Bombus fervidus* | *Thoracobombus* | 90 | Logan, UT USA | 8-Jul-2013 | H. Koch | MDVG00000000 |
| Gris2-3-4 | *Bombus griseocollis* | *Cullumanobombus* | 62 | New Haven, CT USA | 8-Jun-2013 | H. Koch | MDUY00000000 |
| Gris1-3 | *Bombus griseocollis* | *Cullumanobombus* | 58 | New Haven, CT USA | 8-Jun-2013 | H. Koch | MDVH00000000 |
| Gris1-6 | *Bombus griseocollis* | *Cullumanobombus* | 50 | New Haven, CT USA | 8-Jun-2013 | H. Koch | MDVI00000000 |
| Gris3-4 | *Bombus griseocollis* | *Cullumanobombus* | 48 | New Haven, CT USA | 8-Jun-2013 | H. Koch | MDVJ00000000 |
| Snod2-1-5 | *Bombus impatiens* | *Pyrobombus* | 62 | New Haven, CT USA | 20-Aug-2012 | H. Koch | MDVA00000000 |
| Nev3CBA3 | *Bombus nevadensis* | *Bombias* | 131 | Logan, UT USA | 7-Jul-2013 | H. Koch | MEIS00000000 |
| Nev4-2 | *Bombus nevadensis* | *Bombias* | 90 | Logan, UT USA | 7-Jul-2013 | H. Koch | MEIR00000000 |
| Occ4-2 | *Bombus occidentalis* | *Bombus* | 54 | Logan, UT USA | 7-Jul-2013 | H. Koch | MEIQ00000000 |
| HK3 | *Bombus pensylvanicus* | *Thoracobombus* | 74 | BFL, Austin TX USA | 2-Jun-2013 | H. Koch | MEIU00000000 |
| HK9x | *Bombus pensylvanicus* | *Thoracobombus* | 110 | BFL, Austin TX USA | 2-Jun-2013 | H. Koch | MEIT00000000 |
| Pens2-2-5 | *Bombus pensylvanicus* | *Thoracobombus* | 132 | BFL, Austin TX, USA | 2-Jun-2013 | H. Koch | MDUZ00000000 |
| WF3-3 | *Bombus pensylvanicus* | *Thoracobombus* | 67 | BFL, Austin TX USA | 2-Jun-2013 | H. Koch | MEIO00000000 |
| Ruf1-X | *Bombus rufocinctus* | *Cullumanobombus* | 31 | New Haven, CT USA | 8-Jun-2013 | H. Koch | MEIP00000000 |
| wkB29 | *Bombus vagans* | *Pyrobombus* | 55 | New Haven, CT USA | 17-Jun-11 | W. Kwong | [11] |
