# Supplementary figures and images for: Diversification of Type VI Secretion System Toxins Reveals Ancient Antagonism among Bee Gut Microbes
Source: mBio. 2017 Dec 12;8(6):e01630-17. doi: 10.1128/mBio.01630-17 (PMC5727410; doi:10.1128/mBio.01630-17)

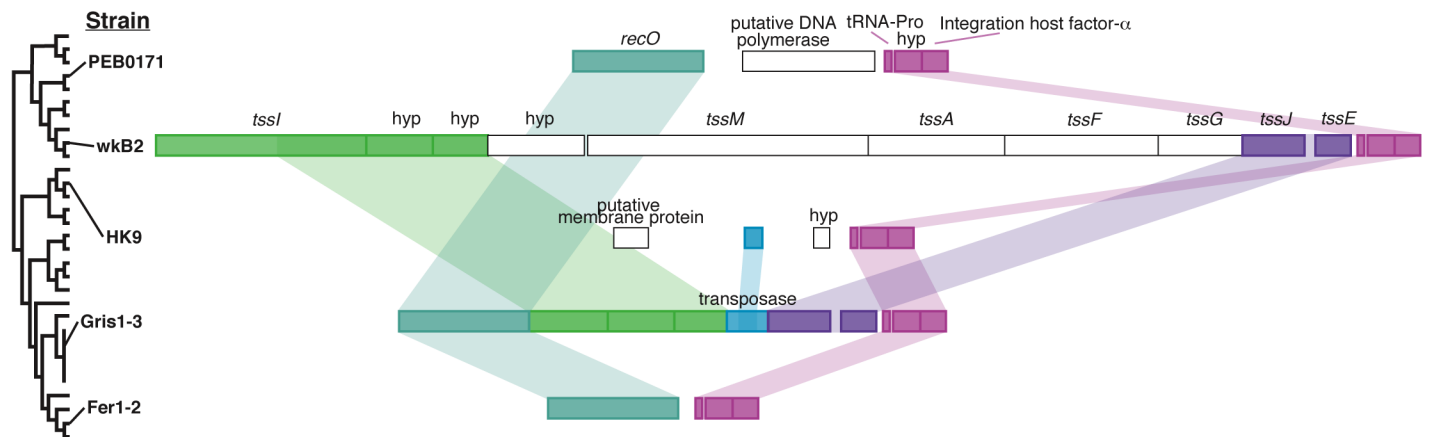

Supplement: FIG S1 [file mbo006173631sf1.pdf]

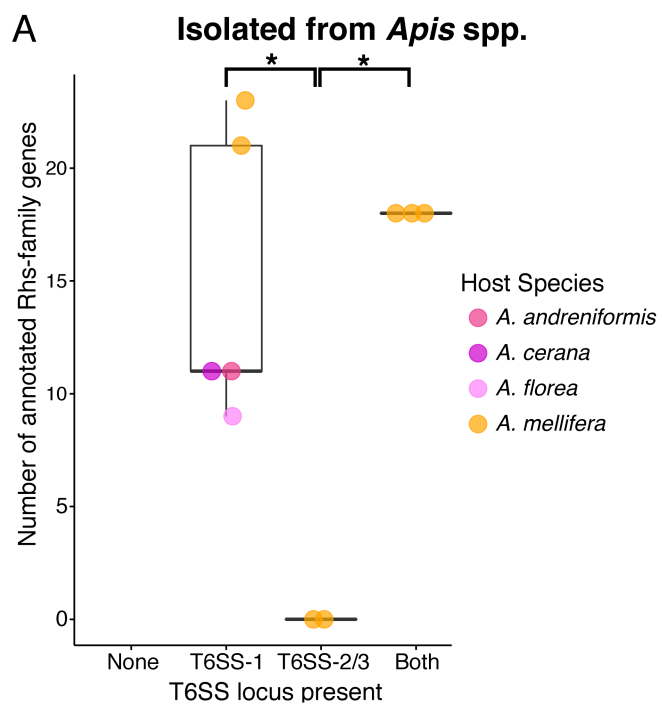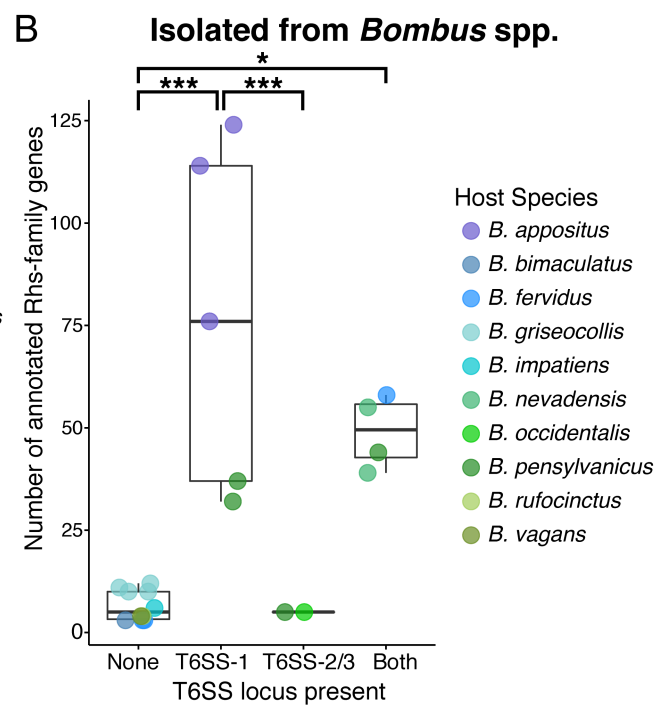

Supplement: FIG S2 [file mbo006173631sf2.pdf]

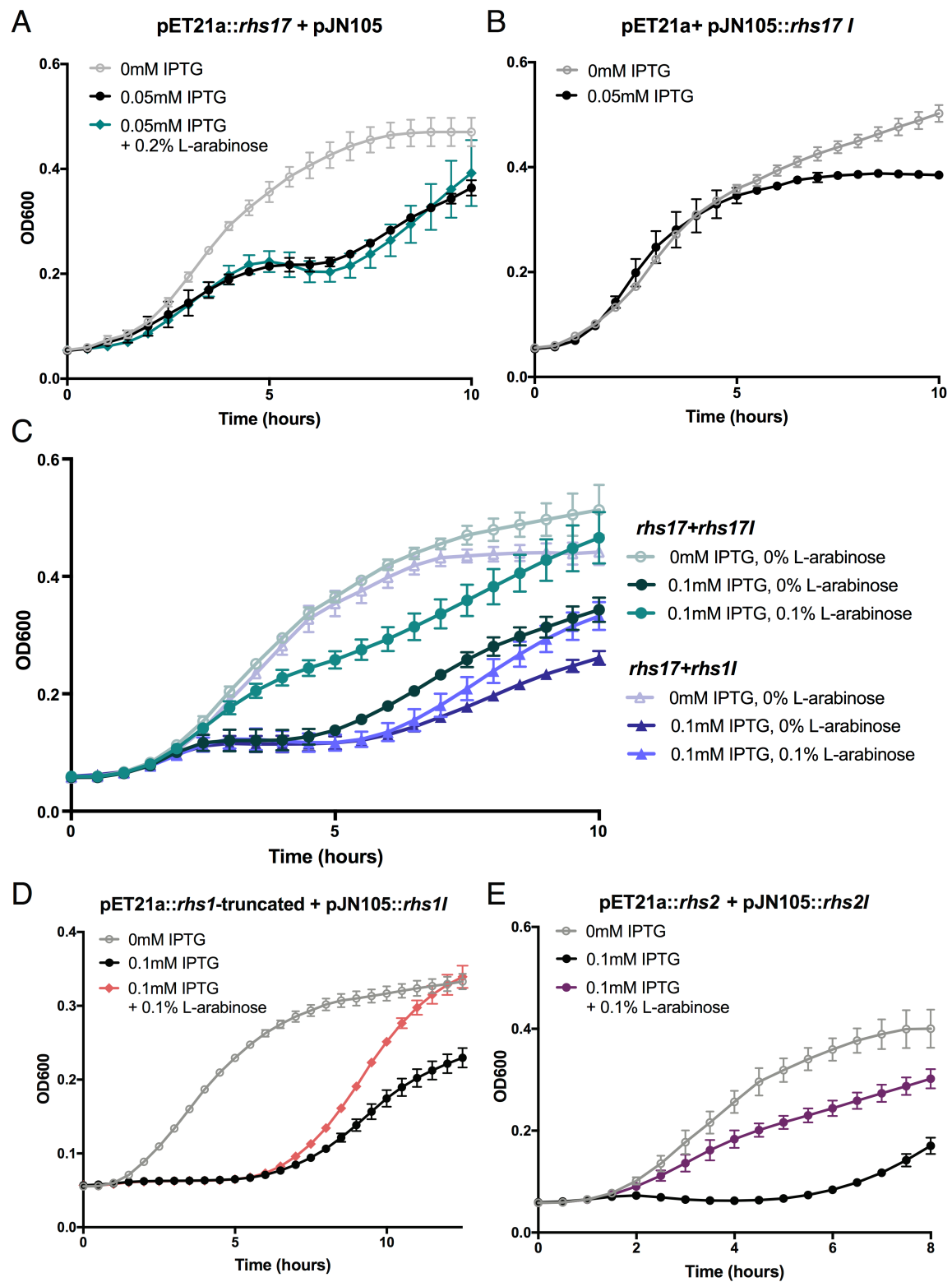

Supplement: FIG S3 [file mbo006173631sf3.pdf]

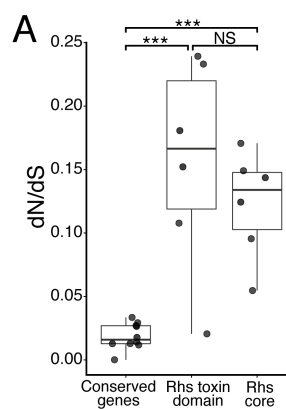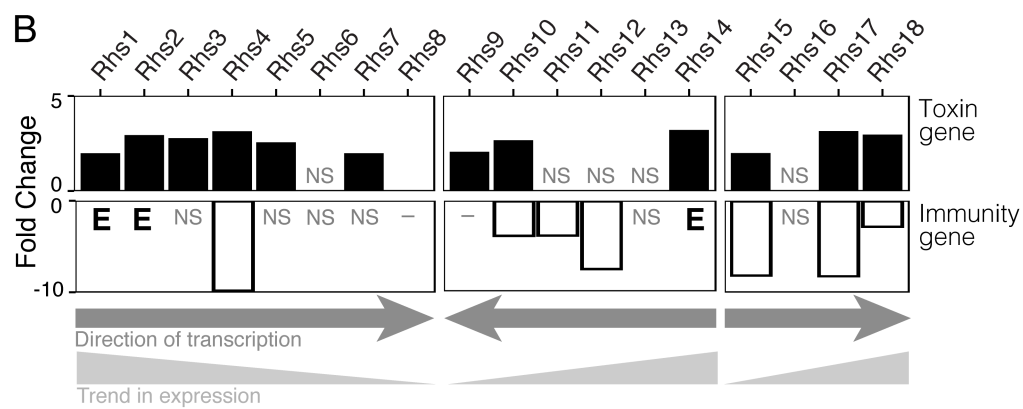

Supplement: FIG S4 [file mbo006173631sf4.pdf]
