# Supplementary material for: Diversification of Type VI Secretion System Toxins Reveals Ancient Antagonism among Bee Gut Microbes
Source: mBio. 2017 Dec 12;8(6):e01630-17. doi: 10.1128/mBio.01630-17 (PMC5727410; doi:10.1128/mBio.01630-17)
Supplement: TABLE S3 [file mbo006173631st3.docx]

**Table S3.** *S. alvi* genes used to reconstruct strain phylogeny and genes used for tests of positive selection.

| **Genes used to reconstruct**  ***S. alvi* strain phylogeny** |  | **Genes used in tests of selection** | | |
| --- | --- | --- | --- | --- |
|  |  | **Highly conserved genes** | **Rhs core regions** | **Rhs C-terminal domains** |
| LSU ribosomal protein L1p (10Ae) |  | 50S ribosomal protein L1 | Rhs4 | Rhs1 |
| LSU ribosomal protein L2p (L8e) |  | 50S ribosomal protein L2 | Rhs6 | Rhs3 |
| LSU ribosomal protein L3p (L3e) |  | 50S ribosomal protein L3 | Rhs10 | Rhs6 |
| LSU ribosomal protein L4p (L1e) |  | 50S ribosomal protein L6 | Rhs11 | Rhs13 |
| LSU ribosomal protein L5p (L11e) |  | 50S ribosomal protein L13 | Rhs13 | Rhs15 |
| LSU ribosomal protein L6p (L9e) |  | 50S ribosomal protein L25 | Rhs16 | Rhs16 |
| LSU ribosomal protein L7/L12 (P1/P2) |  | 30S ribosomal protein S1 |  |  |
| LSU ribosomal protein L13p (L13Ae) |  | 30S ribosomal protein S2 |  |  |
| LSU ribosomal protein L15p (L27Ae) |  | 30S ribosomal protein S4 |  |  |
| LSU ribosomal protein L16 (L10e) |  | Phenylalanyl-tRNA synthetase alpha chain | |  |
| LSU ribosomal protein L17p |  |  |  |  |
| LSU ribosomal protein L18p (L5e) |  |  |  |  |
| LSU ribosomal protein L19p |  |  |  |  |
| LSU ribosomal protein L20p |  |  |  |  |
| LSU ribosomal protein L21p |  |  |  |  |
| LSU ribosomal protein L22p (L17e) |  |  |  |  |
| LSU ribosomal protein L23p (L23Ae) |  |  |  |  |
| LSU ribosomal protein L24p (L26Ae) |  |  |  |  |
| LSU ribosomal protein L25p |  |  |  |  |
| LSU ribosomal protein L27p |  |  |  |  |
| LSU ribosomal protein L28p |  |  |  |  |
| LSU ribosomal protein L30p (L7e) |  |  |  |  |
| LSU ribosomal protein L32p |  |  |  |  |
| SSU ribosomal protein S1p |  |  |  |  |
| SSU ribosomal protein S2p (Sae) |  |  |  |  |
| SSU ribosomal protein S3p (S3e) |  |  |  |  |
| SSU ribosomal protein S4p (S9e) |  |  |  |  |
| SSU ribosomal protein S5p (S2e) |  |  |  |  |
| SSU ribosomal protein S8p (S15Ae) |  |  |  |  |
| SSU ribosomal protein S9p (S16e) |  |  |  |  |
| SSU ribosomal protein S10p (S20e) |  |  |  |  |
| SSU ribosomal protein S11p (S14e) |  |  |  |  |
| SSU ribosomal protein S13p (S18e) |  |  |  |  |
| SSU ribosomal protein S14p (S29e) |  |  |  |  |
| SSU ribosomal protein S16p |  |  |  |  |
| SSU ribosomal protein S17p (S11e) |  |  |  |  |
| SSU ribosomal protein S19p (S15e) |  |  |  |  |
