# Supplementary material for: Diversification of Type VI Secretion System Toxins Reveals Ancient Antagonism among Bee Gut Microbes
Source: mBio. 2017 Dec 12;8(6):e01630-17. doi: 10.1128/mBio.01630-17 (PMC5727410; doi:10.1128/mBio.01630-17)
Supplement: TABLE S4 [file mbo006173631st4.docx]

**Table S4.** Conserved protein domains detected in *S. alvi* wkB2 Rhs toxin genes (e-value < 0.00001) and immunity genes (e-value < 0.001).

| **Category** | **Protein** | **Domain** | **Accession** | **Interval** | **e-value*** |
| --- | --- | --- | --- | --- | --- |
| Toxin | Rhs1 | PAAR_RHS | cd14742 | 328-795 | 7.67E-17 |
|  |  | RhsA | COG3209 | 1867-4125 | 5.56E-65 |
|  |  | PRK13875 | PRK13875 | 67-273 | 9.12E-06 |
| Immunity | Rhs1I |  |  |  |  |
| Toxin | Rhs2 | RhsA | COG3209 | 43-873 | 4.11E-30 |
| Immunity | Rhs2I | SMI1_KNR4 | smart00860 | 124-393 | 5.34E-04 |
| Toxin | Rhs3 | Tox-GHH | pfam15636 | 928-1164 | 1.90E-12 |
|  |  | RhsA | COG3209 | 160-1212 | 2.58E-26 |
|  |  | Ntox30 | pfam15532 | 1123-1389 | 4.81E-07 |
| Immunity | Rhs3I |  |  |  |  |
| Toxin | Rhs4 | RhsA | COG3209 | 94-756 | 2.64E-27 |
|  |  | Tox-ART-HYD1 | pfam15633 | 778-987 | 6.65E-06 |
| Immunity | Rhs4I |  |  |  |  |
| Toxin | Rhs5 | RhsA | COG3209 | 133-1191 | 1.98E-30 |
| Immunity | Rhs5I | Gpos_tandem_5TM | TIGR01218 | 4-273 | 7.45E-07 |
| Toxin | Rhs6 | RhsA | COG3209 | 58-390 | 2.17E-22 |
| Immunity | Rhs6I |  |  |  |  |
| Toxin | Rhs7 | RhsA | COG3209 | 4-1068 | 2.52E-31 |
| Immunity | Rhs7I |  |  |  |  |
| Toxin | Rhs8 | RhsA | COG3209 | 1-1041 | 1.04E-26 |
| Toxin | Rhs9 | RhsA | COG3209 | 82-531 | 2.27E-08 |
| Toxin | Rhs10 | RhsA | COG3209 | 346-810 | 5.63E-25 |
| Immunity | Rhs10I | Cys_rich_CPCC | pfam14206 | 109-207 | 7.79E-05 |
| Toxin | Rhs11 | RhsA | COG3209 | 220-789 | 1.57E-24 |
| Immunity | Rhs11I |  |  |  |  |
| Toxin | Rhs12 | RhsA | COG3209 | 361-861 | 1.04E-17 |
| Immunity | Rhs12I | COX2 | MTH00038 | 83-220 | 1.25E-04 |
| Toxin | Rhs13 | RhsA | COG3209 | 322-849 | 2.33E-25 |
| Immunity | Rhs13I |  |  |  |  |
| Toxin | Rhs14 | RhsA | COG3209 | 325-2574 | 4.09E-66 |
|  |  | PAAR_RHS | cd14742 | 3655-3840 | 2.10E-15 |
| Immunity | Rhs14I |  |  |  |  |
| Toxin | Rhs15 | PAAR_RHS | cd14742 | 268-732 | 2.59E-17 |
|  |  | RhsA | COG3209 | 1807-4059 | 1.01E-66 |
|  |  | Tox-HNH-EHHH | pfam15657 | 4150-4341 | 2.91E-10 |
| Immunity | Rhs15I | AAA_15 | pfam13175 | 16-501 | 5.05E-07 |
| Toxin | Rhs16 | RhsA | COG3209 | 79-657 | 9.72E-23 |
| Immunity | Rhs16I | PRK13979 | PRK13979 | 13-309 | 8.94E-04 |
| Toxin | Rhs17 | RhsA | COG3209 | 7-1287 | 6.26E-35 |
|  |  | LHH | pfam14411 | 1189-1434 | 8.40E-37 |
| Immunity | Rhs17I | SMI1_KNR4 (SUKH-1) | pfam09346 | 73-429 | 8.65E-19 |
| Toxin | Rhs18 | RhsA | COG3209 | 43-702 | 1.09E-27 |
| Immunity | Rhs18I | glyco_rpt_poly | TIGR04370 | 6-293 | 1.15E-04 |

*Based on NCBI conserved domains database [30].
