# Supplementary material for: Diversification of Type VI Secretion System Toxins Reveals Ancient Antagonism among Bee Gut Microbes
Source: mBio. 2017 Dec 12;8(6):e01630-17. doi: 10.1128/mBio.01630-17 (PMC5727410; doi:10.1128/mBio.01630-17)
Supplement: TABLE S5 [file mbo006173631st5.docx]

**Table S5.** Fitness effects of transposon insertions in *S. alvi* wkB2 T6SS, Rhs toxin, and putative immunity genes.

| **Category** | **Gene name** | **New Gene ID** | **Essentiality*** | **log_2_ fold change** | **p_adj_** | **Mean normalized counts** |
| --- | --- | --- | --- | --- | --- | --- |
| Immunity | *rhs1I* | SALWKB2_RS00615 | Reduced | -0.836 | 0.57875 | 2.8 |
| Immunity | *rhs2I* | SALWKB2_RS00640 | Reduced | -1.306 | 0.30019 | 5.4 |
| Immunity | *rhs3I* | SALWKB2_RS00660 | Unchanged | 0.765 | 0.41774 | 162.6 |
| Immunity | *rhs4I* | SALWKB2_RS00675 | Unchanged | -3.306 | 0.00013 | 15.1 |
| Immunity | *rhs5I* | SALWKB2_RS00685 | Unchanged | 2.108 | 0.07076 | 103.1 |
| Immunity | *rhs6I* | SALWKB2_RS00700 | Unchanged | 0.504 | 0.30859 | 177.7 |
| Immunity | *rhs10I* | SALWKB2_RS06365 | Unchanged | -1.966 | 0.01756 | 20.3 |
| Immunity | *rhs11I* | SALWKB2_RS06375 | Unchanged | -1.959 | 0.03759 | 57.2 |
| Immunity | *rhs12I* | SALWKB2_RS06385 | Unchanged | -2.913 | 0.00046 | 17.6 |
| Immunity | *rhs13I* | SALWKB2_RS06410_new | Unchanged | -0.054 | 0.96703 | 63.4 |
| Immunity | *rhs14I* | SALWKB2_RS06425 | Reduced | -0.540 | 0.75248 | 1.9 |
| Immunity | *rhs15I* | SALWKB2_RS10100 | Unchanged | -3.044 | 0.00191 | 10.6 |
| Immunity | *rhs16I* | SALWKB2_RS10110 | Unchanged | 1.180 | 0.11019 | 187.1 |
| Immunity | *rhs17I* | SALWKB2_RS10120 | Unchanged | -3.061 | 0.00064 | 20.4 |
| Immunity | *rhs18I* | SALWKB2_RS10130 | Unchanged | -1.546 | 0.00245 | 75.8 |
| Immunity | *rhs7I* | SALWKB2_RS11525 | Unchanged | -1.040 | 0.37306 | 31.6 |
| T6SS | *tssD* | SALWKB2_RS09760 | Unchanged | 0.060 | 0.94405 | 139.0 |
| T6SS-1 | *tssA* | SALWKB2_RS00515 | Unchanged | 0.765 | 0.09990 | 1517.5 |
| T6SS-1 | *tssG* | SALWKB2_RS00520 | Unchanged | 0.849 | 0.03415 | 759.8 |
| T6SS-1 | *tssF* | SALWKB2_RS00525 | Unchanged | 1.140 | 0.09673 | 4043.5 |
| T6SS-1 | *tssE* | SALWKB2_RS00530 | Unchanged | -0.117 | 0.86118 | 927.2 |
| T6SS-1 | hyp | SALWKB2_RS00535 | Unchanged | 1.344 | 0.07888 | 1923.0 |
| T6SS-1 | hyp | SALWKB2_RS00540 | Unchanged | 2.161 | 0.00010 | 2283.0 |
| T6SS-1 | *tssH* | SALWKB2_RS00545 | Unchanged | 2.338 | 0.00537 | 1475.8 |
| T6SS-1 | *tssB* | SALWKB2_RS00550 | Unchanged | 2.433 | 0.00001 | 1183.8 |
| T6SS-1 | *tssC* | SALWKB2_RS00555 | Unchanged | 1.131 | 0.04154 | 1777.5 |
| T6SS-1 | *tssD* | SALWKB2_RS00560 | Unchanged | 1.785 | 0.00544 | 1275.2 |
| T6SS-1 | *tssJ* | SALWKB2_RS00565 | Unchanged | 1.307 | 0.06431 | 375.2 |
| T6SS-1 | *tssK* | SALWKB2_RS00570 | Unchanged | 0.556 | 0.05952 | 1836.5 |
| T6SS-1 | *tssL* | SALWKB2_RS00575 | Unchanged | 1.222 | 0.04699 | 513.3 |
| T6SS-1 | hyp | SALWKB2_RS00580 | Unchanged | 1.046 | 0.00922 | 1016.3 |
| T6SS-1 | *tssM* | SALWKB2_RS00585 | Unchanged | 1.005 | 0.01669 | 3638.3 |
| T6SS-1 | hyp | SALWKB2_RS00590 | Unchanged | -0.032 | 0.95712 | 455.1 |
| T6SS-1 | hyp | SALWKB2_RS00595 | Unchanged | 2.519 | 0.04208 | 413.4 |
| T6SS-1 | *tssI* | SALWKB2_RS00600 | Unchanged | 2.254 | 0.00026 | 3602.0 |
| T6SS-2 | *tssI* | SALWKB2_RS04595 | Unchanged | 0.815 | 0.05449 | 352.4 |
| T6SS-2 | hyp | SALWKB2_RS04605 | Unchanged | 1.131 | 0.00059 | 543.9 |
| T6SS-2 | hyp | SALWKB2_RS04610 | Unchanged | 1.902 | 0.00057 | 951.5 |
| T6SS-2 | *tssM* | SALWKB2_RS04615 | Unchanged | 0.698 | 0.47385 | 601.5 |
| T6SS-2 | *tssA* | SALWKB2_RS04620 | Unchanged | 2.443 | 0.00000 | 1003.1 |
| T6SS-2 | *tssG* | SALWKB2_RS04630 | Unchanged | 0.347 | 0.81812 | 36.7 |
| T6SS-2 | *tssJ* | SALWKB2_RS04635 | Unchanged | 0.185 | 0.87355 | 36.3 |
| T6SS-3 | *tssH* | SALWKB2_RS06900 | Unchanged | 0.715 | NA | 471.4 |
| T6SS-3 | *tssD* | SALWKB2_RS06905 | Unchanged | 0.007 | 0.99498 | 38.3 |
| T6SS-3 | *tssK* | SALWKB2_RS06920 | Unchanged | 1.434 | 0.00077 | 435.7 |
| T6SS-3 | hyp | SALWKB2_RS06925 | Unchanged | -0.264 | 0.72415 | 108.5 |
| T6SS-3 | *tssC* | SALWKB2_RS06930 | Unchanged | -0.065 | 0.94391 | 179.7 |
| T6SS-3 | *tssB* | SALWKB2_RS06935 | Unchanged | 1.140 | 0.10994 | 54.4 |
| Toxin | *rhs1* | SALWKB2_RS00610 | Unchanged | 0.988 | 0.00007 | 1606.5 |
| Toxin | *rhs2* | SALWKB2_RS00630_new | Unchanged | 1.558 | 0.00760 | 1259.2 |
| Toxin | *rhs3* | SALWKB2_RS00650_new | Unchanged | 1.474 | 0.00080 | 1631.6 |
| Toxin | *rhs4* | SALWKB2_RS00665_new | Unchanged | 1.649 | 0.00000 | 2351.9 |
| Toxin | *rhs5* | SALWKB2_RS00680_new | Unchanged | 1.357 | 0.00018 | 1434.3 |
| Toxin | *rhs6* | SALWKB2_RS00695_new | Unchanged | 1.116 | 0.08450 | 1001.4 |
| Toxin | *rhs7* | SALWKB2_RS00705_new | Unchanged | 0.985 | 0.00052 | 1190.9 |
| Toxin | *rhs8* | SALWKB2_RS00720_new | Unchanged | 0.750 | 0.34557 | 540.9 |
| Toxin | *rhs9* | SALWKB2_RS06340_new | Unchanged | 1.046 | 0.00260 | 184.8 |
| Toxin | *rhs10* | SALWKB2_RS06366 | Unchanged | 1.419 | 0.00407 | 550.5 |
| Toxin | *rhs11* | SALWKB2_RS06376 | Unchanged | 1.035 | 0.15028 | 500.0 |
| Toxin | *rhs12* | SALWKB2_RS06395_new | Unchanged | 0.021 | 0.95910 | 260.0 |
| Toxin | *rhs13* | SALWKB2_RS06415_new | Unchanged | 1.074 | 0.11019 | 863.2 |
| Toxin | *rhs14* | SALWKB2_RS06430 | Unchanged | 1.682 | 0.00004 | 3672.6 |
| Toxin | *rhs15* | SALWKB2_RS10095 | Unchanged | 0.996 | 0.00000 | 1532.5 |
| Toxin | *rhs16* | SALWKB2_RS10105_new | Unchanged | 0.539 | 0.51299 | 479.1 |
| Toxin | *rhs17* | SALWKB2_RS10119 | Unchanged | 1.654 | 0.00035 | 2001.8 |
| Toxin | *rhs18* | SALWKB2_RS10125_new | Unchanged | 1.564 | 0.00000 | 1198.0 |

*Genes were considered essential if mutants with insertions in these genes were significantly less abundant in

the control treatment than could be expected by chance, as described in [31].

p_adj_, Benjamini-Hochberg adjusted p-values.
