# Supplementary material for: Diversification of Type VI Secretion System Toxins Reveals Ancient Antagonism among Bee Gut Microbes
Source: mBio. 2017 Dec 12;8(6):e01630-17. doi: 10.1128/mBio.01630-17 (PMC5727410; doi:10.1128/mBio.01630-17)
Supplement: TABLE S6 [file mbo006173631st6.docx]

**Table S6.** Primers used to clone Rhs toxin and immunity genes into expression vectors.

| **Primer** | **Sequence** | **Target Gene** | **Vector** | **Cloning method** |
| --- | --- | --- | --- | --- |
| Rhs1-trunc_NdeI_FW | GGAATTCCATATGGAAACCGGCTTACATTACAATACG | *rhs1* | pET21a | Restriction cloning |
| Rhs1_XhoI_RV | ATGGCTCGAGTTATTTTCTCCCACATACCTTGC |  |  |  |
| Rhs2_NdeI_FW | GGAATTCCATATGCTGTTGGTAAGCAAAAAGCA | *rhs2* | pET21a | Restriction cloning |
| Rhs2_NotI_RV | ATGGGCGGCCGCTTATCAGCATCGTTGTCTTAAAGTAG |  |  |  |
| pET21a_rhs17_Gibs_FW | AGAAATAATTTTGTTTAACTTTAAGAAGGAGATATACAATGATACATCGCGACAACCTGC | *rhs17* | pET21a | Gibson assembly |
| pET21a_rhs17_Gibs_RV | CCCATTTGCTGTCCACCAGTCATGCTAGCCATTATCACATAAAATCCAATGCTCTTAATT |  |  |  |
| pJN105_Rhs1I_FW2 | ACCCGTTTTTTTGGGCTAGCGAATGCAATCATTTATATGGATTTTTCATG | *rhs1I* | pJN105 | Gibson assembly |
| pJN105_Rhs1I_RV2 | CGTAATACGACTCACTATAGGGCGAATTGTTATTCCATCTTCTCCCCATTTTG |  |  |  |
| Rhs2I_EcoRI_FW | GGAATTCATGTTTATTGATGATGCTTTACG | *rhs2I* | pJN105 | Restriction cloning |
| Rhs2I_SacI_RV | CGCAACGAGCTCTTATCCAAAGAAAAAATACATAAAATTATC |  |  |  |
| pJN105_rhs17I_Gibs_FW | AGCGAATTCCTGCAGCCCGGGGGATCCACTAGTTATGGGTGATATTAAAAATCTTGTTAC | *rhs17I* | pJN105 | Gibson assembly |
| pJN105_rhs17I_Gibs_RV | GCGAATTGGAGCTCCACCGCGGTGGCGGCCGCTTTACGTCCGTAAGTTTACATATTCTTC |  |  |  |
